# Eliminating Resistance–Capacitance Coupling Shielding for Depicting the Defect Landscape in Perovskite Solar Cells by Capacitance Spectroscopy

**DOI:** 10.1002/advs.202403984

**Published:** 2024-06-19

**Authors:** Biao Li, Daoyong Zhang, Zhenyi Ni, Pengjie Hang, Yuxin Yao, Chenxia Kan, Xuegong Yu, Deren Yang

**Affiliations:** ^1^ State Key Laboratory of Silicon and Advanced Semiconductor Materials and School of Materials Science and Engineering Zhejiang University Hangzhou 310027 China; ^2^ ZJU‐Hangzhou Global Scientific and Technological Innovation Center Hangzhou 311200 China

**Keywords:** defects, perovskite solar cells, thermal admittance spectroscopy, trap states

## Abstract

Capacitance spectroscopy techniques have been widely utilized to evaluate the defect properties in perovskites, which contribute to the efficiency and operation stability development for perovskite solar cells (PSCs). Yet the interplay between the charge transporting layer (CTL) and the perovskite on the capacitance spectroscopy results is still unclear. Here, they show that a pseudo‐trap‐state capacitance signal is generated in thermal admittance spectroscopy (TAS) due to the enhanced resistance capacitance (RC) coupling caused by the carrier freeze‐out of the CTL in PSCs, which could be discerned from the actual defect‐induced trap state capacitance signal by tuning the series resistance of PSCs. By eliminating the RC coupling shielding effect on the defect‐induced capacitance spectroscopy, it is obtain the actual defect density which is 4‐folds lower than the pseudo‐trap density, and the spatial distribution of defects in PSCs which reveals that the commonly adopted interface passivators can passivate the defects about 60 nm away from the decorated surface. It is further revealed that phenethylammonium ions (PEA^+^) possess a better passivation capability over octylammonium ions (OA^+^) due to the deeper passivation depth for PEA^+^ on the surface defects in perovskite films.

## Graphical abstract

Here, it is found that a pseudo‐trap‐state capacitance signal induced by resistance capacitance coupling shielding effect can be discerned from the actual defect‐induced trap‐state capacitance signal by tuning the series resistance of perovskite solar cells (PSCs). A method is established for the first time to eliminate the resistance capacitance coupling shielding effect to obtain the actual defect‐induced trap densities in PSCs.

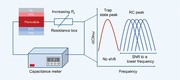

## 1 Introduction

Perovskite solar cells (PSCs) have progressed astonishingly in the past decade, achieving a power conversion efficiency >26% which is comparable to commercial silicon solar cells.^[^ ^1^ ^]^ The rocketing development of PSCs takes credit to the understanding and management of defects, especially those acting as trap states with strong electrical activity in perovskite films and their interfaces which induce non‐radiative recombination losses in PSCs.^[^ ^2^, ^3^, ^4^, ^5^, ^6^, ^7^ ^]^ Knowing the whole landscape of trap states in perovskites including their energy and spatial distributions as well as their defect nature has become an important task that will contribute to a better management of the defects in perovskites to further improve their solar cell performances.^[^ ^8^ ^]^

Capacitance‐based techniques, such as deep‐level transient spectroscopy (DLTS), drive‐level capacitance profiling (DLCP), and thermal admittance spectroscopy (TAS) are powerful techniques to investigate trap states in PSCs.^[^ ^8^, ^9^, ^10^ ^]^ Among these techniques, TAS has now become one of the most frequently adopted methods in PSCs given its capability for generating critical defect information such as defect density, energy depth and capturing cross‐section of trap states in PSCs and simplicity for operation via frequency‐dependent capacitance scans.^[^ ^8^, ^10^, ^11^, ^12^ ^]^ However, the reliability of TAS method operated at high and low‐frequency domains for PSCs is still questioned and is believed to be subjected to the presence of charge transport layers and ion migrations in PSCs.^[^ ^12^, ^13^ ^]^ Almora et al. reported a temperature‐independent capacitance signature at high‐frequency domains in TAS results and speculated it to be caused by capacitance resistance effect.^[^ ^14^ ^]^ Awni et al. recently revealed that hole transport layers (HTLs) introduced TAS signals in PSCs at temperatures below 240 K, which hindered the evaluation of the actual trap densities in PSCs.^[^ ^12^ ^]^ To date, the origin of the series resistance‐related and HTL‐related capacitance signature in PSCs is not clear, and there still lack of effective methods to discern these “fake” capacitance signatures from the defect‐induced trap‐state capacitance signatures in PSCs to help evaluating the actual trap density and energy depth of defects in perovskites. What's more concerning, TAS method is still not readily applied to PSCs to unveil the spatial distribution of trap states in perovskite films due to the lack of understanding on the impact of external biases on the TAS results of PSCs, which greatly limits the broad application of TAS method from being a simple and efficient strategy to manifest the whole landscape of trap states in perovskites.

In this work, we have studied the impacts of commonly adopted HTLs including 220 770‐tetrakis (N,N‐dip‐methoxyphenylamine)−9,90‐spirobifluorene (Spiro) and polytriarylamines (PTAA) on TAS results of PSCs.^[^ ^6^, ^7^, ^15^, ^16^ ^]^ It is revealed that the HTL‐related capacitance signature is caused by the enhanced resistance capacitance (RC) coupling in PSCs due to the carrier freeze‐out of HTLs. The peak of the RC capacitance signature shows a unique series‐resistance (*R*_s_) dependence. We further calibrate the TAS results via a self‐established mathematic method, which unveils the defect‐induced trap density of states (tDOS) and their spatial distributions in perovskite films.

## 2 Results and Discussion

**Figure 1:**
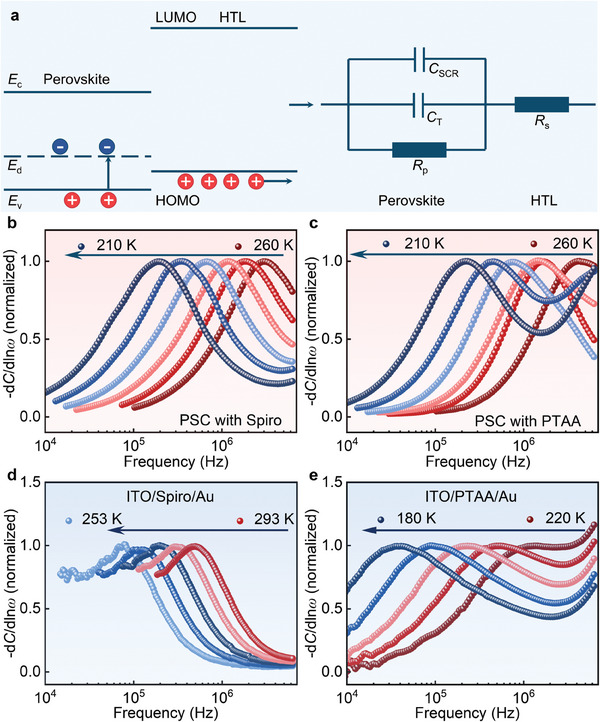
a) The configuration of a n–i–p PSC (left) and its corresponding equivalent circuit (right). *R*_p_ represents the parallel resistance of the PSC. *E*_d_ is the energy depth of the trap states in perovskites. b–e, TAS results for devices with different configurations with a temperature step of 10 K: b) a PSC with Spiro, c) a PSC with PTAA, d) a Spiro only device, e) a PTAA only device.

TAS tests are commonly performed on PSCs with multi‐layers to extract the defect information of perovskites.^[^ ^10^, ^11^, ^17^, ^18^ ^]^ The accuracy of the results is guaranteed when the capacitance of the perovskite layer dominates the total capacitance of the device. In this case, the charge transporting layers could be regarded as *R*_s_ in connection with the space charge capacitance (*C*_SCR_) and trap‐state‐induced capacitance (*C*_T_) of the perovskite layer, as schematically shown by an equivalent circuit of a PSC with a typical n–i–p device structure of ITO/SnO_2_/Perovskite/HTL/Au (**Figure** **1**a).^[^ ^19^, ^20^, ^21^ ^]^ Note that the SnO_2_ layer is not presented in the equivalent circuit given its ultrathin thickness (≈20 nm) and hence large capacitance and rather small resistance. The trapping and de‐trapping of charge carriers by trap states in perovskites cause the changing of *C*_T_, resulting in a characteristic peak in the d*C*/dln*ω* – *ω* plot (where *ω* is the angular frequency of ac signal) which gives the information of the trap density and energy depth (*E*_d_) of the trap state.^[^ ^22^ ^]^

To understand the impacts of HTLs on TAS results of PSCs, we mainly fabricated n–i–p structured PSCs with commonly used HTL materials including Spiro and PTAA (see the Supporting Information).^[^ ^16^, ^23^, ^24^ ^]^ The electron transporting layers (ETL, SnO_2_) and the perovskite layer (formamidinium lead iodide, FAPbI_3_) were kept constant for all the devices. The representative current density–voltage (*J*–*V*) curves of the solar cells are shown in Figure S1 (Supporting Information). The PSC with Spiro shows a typical power conversion efficiency (PCE) of 23.67% at reverse scan mode, which is highly efficient for a n–i–p PSC without any bulk or interface defect passivations for the perovskite films.^[^ ^7^ ^]^ Then we performed TAS measurements on these devices at different temperatures. Interestingly, it is found that the PSCs with Spiro and PTAA exhibit characteristic capacitance peaks in the d*C*/dln*ω* – *ω* plot at frequencies ranging from 10^5^ to 10^6^ Hz (Figure 1b,c; Figure S2a,b, Supporting Information), which is absent in HTL‐free PSCs with device structures of ITO/SnO_2_/Perovskite/Au and ITO/Perovskite/Au (Figure S3, Supporting Information). These results indicate that the measured TAS peaks at frequencies ranging from 10^5^ −10^6^ Hz in PSCs with Spiro and PTAA should not be caused by the perovskite layer. Instead, they are related to the electric properties of the HTLs. To verify the HTL‐induced TAS peaks in these PSCs, we removed the perovskite layer from the solar cell and performed TAS tests on ITO/Spiro/Au and ITO/PTAA/Au devices (Figure S2c,d, Supporting Information). These devices again show similar peaks in the d*C*/dln*ω* – *ω* plot (Figure 1d,e) with the complete structured PSCs. Moreover, the HTL‐related TAS peak shifts to a lower frequency with the decrease of the temperature (Figure 1b–e), with an apparent activation energy of 0.23 eV (Figure S4, Supporting Information), which resembles the characteristic of a trap state in perovskites. It is thus imperative to figure out the origin of this peak and distinguish it from the defect‐induced trap state peak in perovskites.

**Figure 2:**
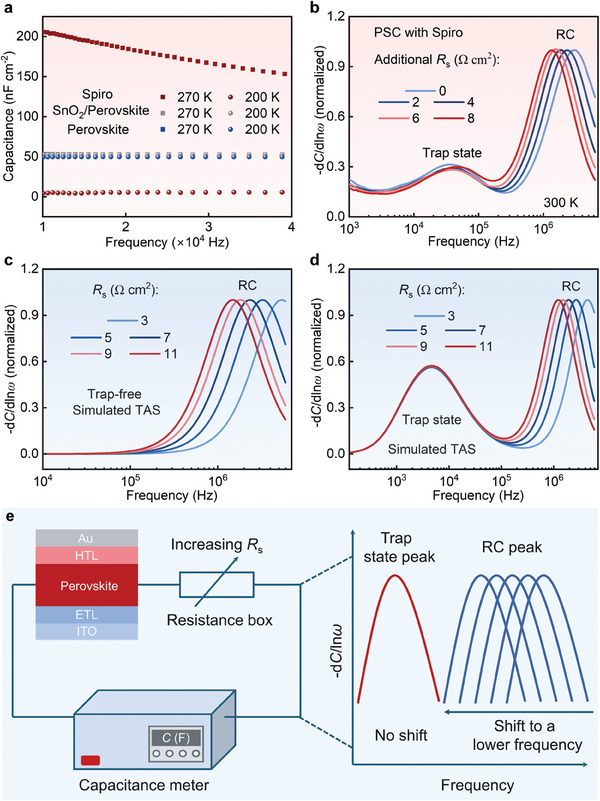
a) Capacitance of different devices at different temperatures with ITO and Au as electrodes. b) Measured TAS results with additional *R*_s_. c) Simulated TAS results for a trap‐free PSC with different *R*_s_. d) Simulated TAS results for a PSC with trap states in perovskite films and different *R*_s_. e) Schematic illustration of the method for discerning RC peak and defect‐induced trap state peak.

To solve this puzzle, we first measured the frequency‐dependent capacitance of HTL‐only (ITO/Spiro/Au), perovskite‐only (ITO/Perovskite/Au), and ETL‐incorporated perovskite (ITO/SnO_2_/Perovskite/Au) devices at temperatures of 270 and 200 K, respectively. It is seen that at 270 K the capacitance of the Spiro device is much larger than the capacitance of the perovskite device with and without the ETL (**Figure** **2**a), indicating that the capacitance of a regular n–i–p PSC is dominated by the capacitance of the perovskite layer at high temperatures. Meanwhile, the incorporation of the ETL hardly changes the capacitance of the perovskite devices, validating the negligible influence of the thin SnO_2_ layer on the capacitance of the PSC (Figure 1a). With the decrease of the temperature, the capacitances of the perovskite devices with and without the ETL remain basically the same. In contrast, the capacitance of the Spiro‐only device reduces significantly at low temperatures (Figure 2a). This implies a much higher temperature sensitivity of the capacitance of Spiro compared to that of perovskites. The capacitance of Spiro could be described by

(1) $$C\;=\;A\sqrt{\frac{\varepsilon_{0}\varepsilon_{r}q^{2}p_{0}}{k_{B}T}}$$

where *A* is the area of the device, *ε*_0_ is the vacuum dielectric constant, *ε*_r_ is the dielectric constant of Spiro, *q* is the element charge and *p*_0_ is the thermal equilibrium hole density of Spiro, *k*_B_ is the Boltzmann constant, *T* is the temperature. Equation (1) suggests that the significantly decreased *C* at 200 K is mainly due to the reduction of *p*_0_ which is commonly known as the carrier freeze‐out of Spiro.^[^ ^25^, ^26^ ^]^

The carrier freeze‐out of Spiro and PTAA would significantly reduce the conductivity of the HTL and subsequently increase the *R*_s_ of PSCs.^[^ ^26^ ^]^ To further verify the increase of *R*_s_, we measured the *J–V* curve of a representative PSC with Spiro at different temperatures and extracted the *R*_s_ (Figure S5 and Table S1, Supporting Information). It is shown that the *R*_s_ of PSC increases from 3.68 to 193.03 Ω cm^2^ as the temperature reduces from 290 to 200 K which greatly limits the performance of the PSC. The increased *R*_s_ couples with the capacitance of the perovskite layer and introduces a TAS peak at *ω*_1_ = 1/*CR*_s._^[^ ^27^ ^]^ To verify the impact of the RC coupling effect on the TAS results, a resistance box was deliberately added in series with the PSC at room temperature to simulate the increased resistance of the HTL at low temperatures. The tested PSC shows two TAS peaks at ac frequencies ≈5 × 10^4^ and 10^6^ Hz, respectively (Figure 2b; Figure S6, Supporting Information). Interestingly, with the increase of the additional *R*_s_, the high‐frequency TAS peak shifts to a lower frequency (Figure 2b), similar to the effect of the carrier freeze‐out of the HTL on the TAS peak of PSCs (Figure 1b–e). The high‐frequency TAS peak not only shifts with the increase of *R*_s_ but also varies with the changing of the capacitance of the PSC caused by a reduced device area (Figure S7, Supporting Information), further demonstrating the RC‐coupling‐induced nature of the high‐frequency TAS peak. Similar results were observed in PTAA PSCs (Figure S8, Supporting Information). We also investigated the influence of Poly (3‐hexylthiophene) (P3HT) on the TAS results of n*–*i*–*p PSCs (Figures S9 and S10, Supporting Information).^[^ ^28^ ^]^ It is found that the RC coupling effect induced by P3HT layers is weaker than that of Spiro and PTAA because the commonly adopted P3HT layers are undoped thin layers.^[^ ^28^ ^]^ While in contrast, the low‐frequency TAS peak does not shift with the tuning of *R*_s_ for the PSCs (Figure 2b), which is expected to be caused by trap states in perovskites because the trapping and de‐trapping processes of charge carriers by trap states are independent of the *R*_s_.

To further validate the above analysis, we performed drift‐diffusion simulations to simulate the TAS of PSCs with a device structure of ITO/SnO_2_/Perovskite/Spiro/Au using the SCAPS‐1D software^[^ ^29^ ^]^ (Note S1 and Tables S2–S4, Supporting Information). The Spiro is assumed to be highly doped in the simulation so it can be treated as a purely ohmic circuit element as suggested in Figure 1a. We performed the photoluminescence tests at *V*_OC_ and 0 V to validate the assumption.^[^ ^30^ ^]^ The calculated quasi‐Fermi level splitting (QFLS) at 0 V is 97 meV lower than that at *V*_OC_ which is consistent with the simulated QFLS(V) results produced by SCAPS (Figure S11, Supporting Information). This result proves that the Spiro of the fabricated device is indeed a purely ohmic circuit element. The simulated *J–V* curve of the modeled perovskite solar cell is shown in Figure S12 (Supporting Information). The modeled trap‐free perovskite solar cell shows a PCE of 27.43% which clearly confirms the rationality of our modeling. We intentionally introduced different *R*_s_ for PSCs with and without trap states in the perovskite layer. It is found that even for a trap‐free PSC, a TAS peak occurs at ac frequencies ≈10^[^ ^6^ ^]^ Hz (Figure 2c; Figure S13, Supporting Information). The peak position shifts to lower frequencies with the increase of the *R*_s_ (Figure 2c; Figure S13, Supporting Information) or the increase of the capacitance of the device (Figure S14, Supporting Information). For the PSC with a trap state in the perovskite layer, a defect‐induced trap state peak appears at the ac frequencies ≈10^4^ Hz in addition to the RC‐induced TAS peak (Figure 2d; Figure S13, Supporting Information). The simulated peak position of the trap states does not change with the increase of the *R*_s_, excellently agreeing with the experimentally measured results for PSCs with trap states (Figure 2b). Therefore, our experimental and simulation results both prove that the RC‐coupling‐induced TAS signals mainly originate from the enhanced *R*_s_ which is caused by the carrier freeze‐out of the HTL in PSCs. More importantly, these results demonstrate the different behaviors between the RC coupling and defect‐induced trap state TAS signals in response to the increase of *R*_s_, which could be utilized as an effective strategy to discern the RC TAS peak from the defect‐induced trap state TAS peak in PSCs as illustrated in Figure 2e.

**Figure 3:**
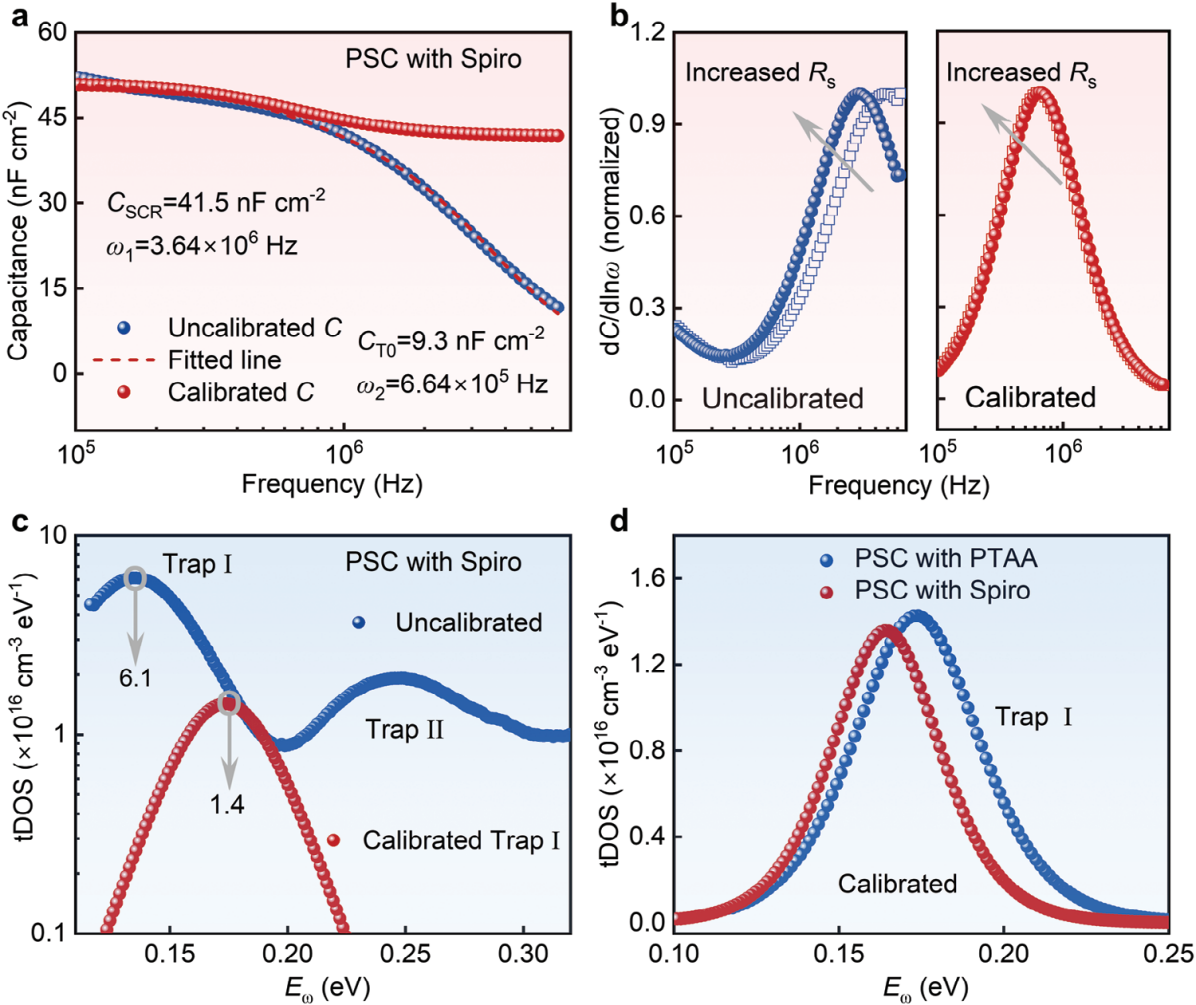
a) Uncalibrated capacitance and calibrated capacitance for PSCs. b) Uncalibrated and calibrated d*C*/dln*ω – ω* plot for the PSC with Spiro and increased additional *R*_s_. c) Uncalibrated tDOS and calibrated tDOS for PSCs with Spiro. d) Calibrated tDOS in PSCs with Spiro and PTAA. Note that the HTL/Perovskite interface of the tested PSC is passivated.

Then we make further efforts to eliminate the influence of RC coupling shielding effect on TAS results to evaluate the actual defect‐induced trap densities in the perovskite films. The total capacitance of a PSC without the influence of the RC coupling effect should be given by *C* = *C*_SCR_ + *C*_T_ (Figure 1a), in which the frequency‐dependent *C*_T_ is described by (Note S2, Supporting Information)^[^ ^31^ ^]^

(2) $$C_{T}=C_{T0}\;\frac{\omega_{2}^{2}}{\omega^{2}+\omega_{2}^{2}}$$

where *ω*_2_ is the characteristic TAS peak frequency of a trap state which is determined by the rate of trapping and de‐trapping of charge carriers by the trap state, and *C*_T0_ is the maximum capacitance induced by the trap state. With the impact of the RC coupling effect, the measured capacitance (*C*_meas_) then becomes^[^ ^27^ ^]^

(3) $$C_{\mathrm{meas}}=\left(C_{\mathrm{SCR}}+C_{T0}\frac{\omega_{2}^{2}}{\omega^{2}+\omega_{2}^{2}}\right)\;\frac{\omega_{1}^{2}}{\omega^{2}+\omega_{1}^{2}}$$

where *ω*_1_ = 1/*CR*_s_ is related to the RC coupling of the device. To obtain the actual defect‐induced tDOS in perovskites, we fitted the uncalibrated *C – ω* curves of PSCs at ac frequencies ranging from 10^5^ to 10^6^ Hz (Figure S6, Supporting Information) with Equation (3) and extracted *ω*_2_ and *C*_T0_ for the calculation of the defect‐induced tDOS with Equation (2). **Figure** **3**a shows that the uncalibrated *C – ω* curve of the PSC with Spiro could be excellently fitted by Equation (3), rendering the calibrated *C – ω* curve without the influence of the RC coupling shielding effect. The calibrated TAS peak in the d*C*/dln*ω – ω* plot stays at the same frequency when the *R*_s_ increases (Figure 3b; Figure S6, Supporting Information), which is in stark difference with the uncalibrated TAS peak, validating the reliability of the established calibration process for eliminating the RC coupling effect for PSCs.

Figure 3c presents the calibrated tDOS of the trap band I of the PSC with Spiro, together with the uncalibrated tDOS of trap bands I and II (Note S3, Figures S6 and S15–S17, Supporting Information). It is noticed that the RC coupling effect leads to a substantial overestimation of the trap density of trap band I, which could be 4 times higher than the defect‐induced trap density (Figure 3c). Meanwhile, the estimated *E*_d_ of trap band I is also shifted by ≈40 meV without the elimination of the RC coupling effect, highlights the importance for the calibration of the tDOS of PSCs to understand the defect‐induced trap density and *E*_d_ in perovskites. Note that the RC coupling effect has negligible influences on the tDOS of trap band II of the PSC. We then compared the defect‐induced tDOS of trap band I in PSCs with Spiro and PTAA (Figures S6 and S8, Supporting Information). Figure 3d shows that the calibrated tDOS is similar between these two solar cells in terms of trap density and *E*_d_ with a diminutive energy deviation smaller than *k*_B_ *T*, which is reasonable since these devices have the same perovskite layers. Moreover, we found that the proposed method can calibrate the *C – ω* curve and eliminate the RC coupling effect for p*–*i*–*n perovskite solar cells (Figure S18, Supporting Information). Then, we attempt to validate the proposed method via drift‐diffusion simulations by SCAPS. We purposely introduce a trap state (*E*_d_ = 0.28 eV) into perovskite films, which can induce a TAS peak in the high‐frequency range of 10^4^ – 10^6^ Hz where RC coupling shielding effect seriously influences the TAS data. The result shows that the simulated *C – ω* data can be well fitted by Equation (3) (Figure S19a, Supporting Information). Figure S19b (Supporting Information) shows that there is no trap state peak with *E*_d_ = 0.28 eV in the uncalibrated tDOS, which suggests that the trap state information is totally covered by the RC coupling shielding effects. However, after calibration, the calculated tDOS result shows a trap state peak with *E*_d_ = 0.28 eV, which further confirms the effectiveness of the established method in calibrating the TAS data.

**Figure 4:**
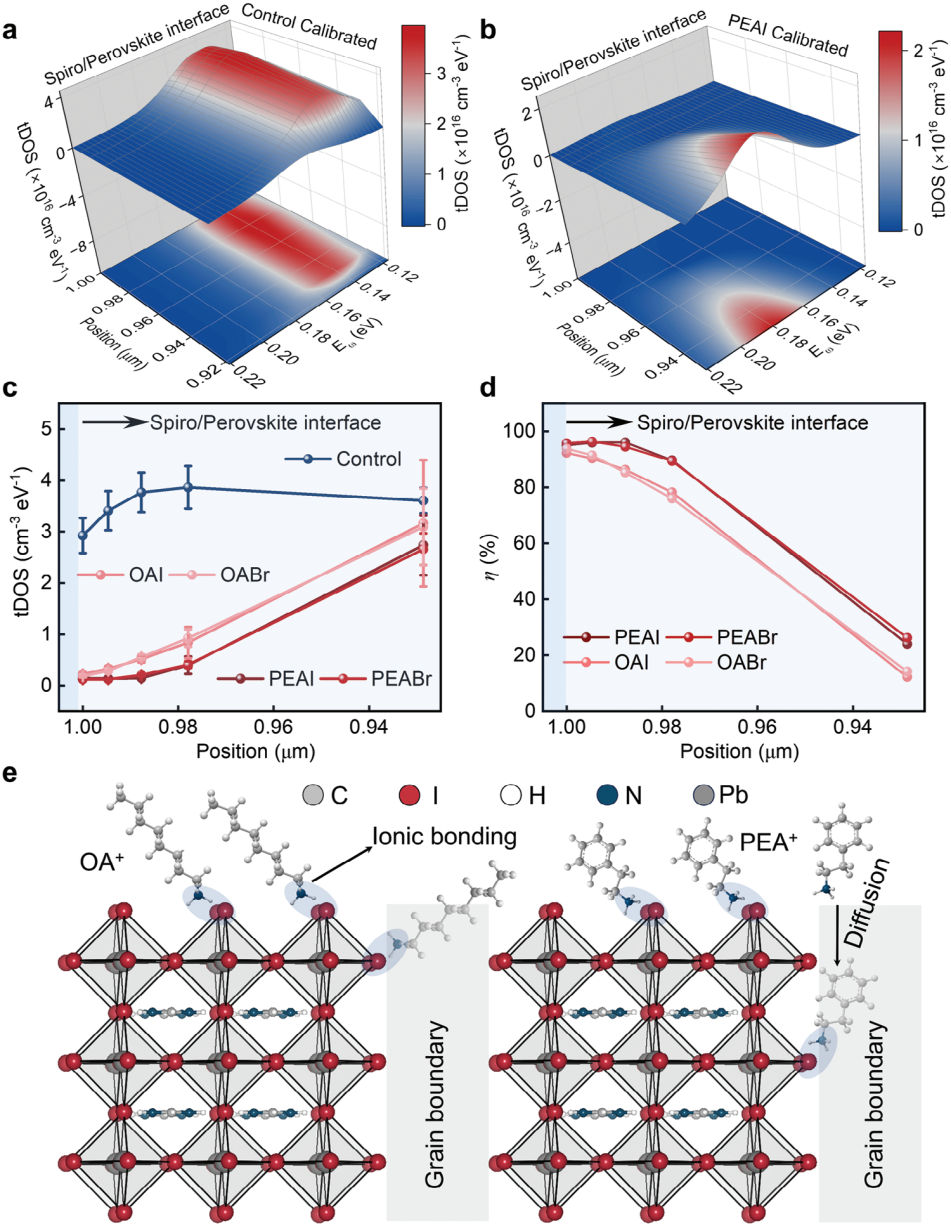
a,b) Pseudo‐color 3D map of TAS results of PSCs and the bottom is the horizontal projection of the 3D map: a) Calibrated tDOS of trap band I in PSCs without interface (HTL/Perovskite) passivationss. b) Calibrated tDOS of trap band I in PSCs with interface passivations by PEAI. c) Calibrated maximum tDOS of trap band I at different positions in three individual PSCs without and with interface passivationss by different passivations agencies. d) Average passivations efficiency for trap band I of different passivations agencies at different positions in three individual PSCs. e) Schematic illustration of the passivations effect of PEA^+^ and OA^+^.

To further locate the defects that cause trap band I in our PSCs, we performed TAS measurements on PSCs at different forward dc biases (0–0.9 V), which changed the depletion width of the solar cell and therefore measured the tDOS of trap band I across the perovskite film.^[^ ^8^, ^17^, ^32^ ^]^ We should mention that this method may lead to inaccurate defect distributions in PSCs since the increased forward bias will cause a significant RC coupling effect via the increased *C*_SCR_. Because the probing of defect distributions of PSCs via capacitance‐based method relies on the sweeping of a depletion region edge across a device from one electrode to the counter electrode, it is critical to understand the location of the junction(s) in the PSCs. We performed cross‐sectional electron beam induced current (EBIC) tests on the fabricated PSC to identify the location of the junction(s) (Note S4, Supporting Information).^[^ ^33^, ^34^ ^]^ The extracted EBIC line scan (Figure S20, Supporting Information) clearly shows that the EBIC decays from the SnO_2_/Perovskite interface to Spiro/Perovskite interface, which indicates that the junction may mainly locates at the SnO_2_/Perovskite interface (Note S4, Supporting Information).^[^ ^33^, ^34^ ^]^ This result verifies that the fabricated perovskite film is of p‐type conductivity (Note S4, Supporting Information) which is consistent with the results in literatures,^[^ ^8^, ^17^ ^]^ and there is a well‐defined depletion region in the fabricated PSCs. Based on these results, we assume that only a primary junction is located at the SnO_2_/Perovskite interface of the fabricated device and then calculated the spatial distribution of defects in perovskites. Therefore, as the forward bias increases, we can gradually modulate the depletion region edge from the Spiro/Perovskite interface to the SnO_2_/Perovskite interface and measure the defect density in the corresponding regions. A typical spatial distribution of trap band I in PSCs measured by TAS at different biases without the elimination of the RC coupling effect is shown in Figure S21 (Supporting Information), which indicates that the density of trap band I increases from the HTL/Perovskite interface to the bulk of the film.^[^ ^35^ ^]^ However, after eliminating the RC coupling effect at forward biases, we find that the trap band I actually mainly exists at the near‐surface (HTL/Perovskite interface) region of the PSC (**Figure** **4**a), which is consistent with previous results.^[^ ^36^, ^37^, ^38^ ^]^ We note that the position (*W*) in Figure 4a is derived by the *ε*_0_ *ε*_r_/*C*_SCR_ at different biases which is deeply correlated with the precise physical position (*x*) of defects.^[^ ^39^ ^]^ Therefore, the variations in *W* correspond to actual variations of *x* over approximately the same distance scale.^[^ ^39^ ^]^ Therefore, it is necessary to revisit the applicability of TAS method at large forward biases considering the strong RC coupling effect in this condition, whereas this work provides a pathway to eliminate the RC coupling effect on the tDOS results and make the TAS method further applicable for the study of the spatial distributions of defects in PSCs.

With the elimination of the RC coupling shielding effect on PSCs at forward biases, we are able to evaluate the effect of interface passivations on the spatial distributions of defects in perovskites simply by using the TAS method. We screened four commonly adopted passivations agencies to passivate the HTL/Perovskite interface, which were phenethylammonium iodide (PEAI), phenylethylammonium bromide (PEABr), octylammonium iodide (OAI), and octylammonium bromide (OABr).^[^ ^40^, ^41^, ^42^, ^43^ ^]^ The parameters of *J–V* curves of these devices are presented in Figures S22 and S23 (Supporting Information). Figure 4b shows the calibrated tDOS of trap band I in perovskite films with PEAI passivations, which clearly shows that PEAI can effectively reduce the density of trap band I from the surface to the bulk of the perovskite film.^[^ ^40^, ^44^ ^]^ A representative PEAI passivated PSC shows a typical PCE of 24.22% at reverse scan mode (Figure S22, Supporting Information), representing an advanced performance for n*–*i*–*p PSCs.^[^ ^7^, ^40^ ^]^ The *C – V* curves and the extracted *V*_bi_ are shown in Figures S24,S25 and Table S5 (Supporting Information). Figure 4c compares the spatial distributions of the maximum tDOS of trap band I from the surface (HTL/Perovskite interface) to the bulk of the perovskite films before and after surface passivations (Figures S26–S28, Supporting Information). It is found that the effect of interface passivations could extend to the bulk of perovskite films with an effective passivations depth of ≈60 nm. They have negligible impacts on the defect of trap band I located deeper than 60 nm from the surface of the perovskite films (Figure 4b,c).

We further defined the passivations efficiency (*η* = (tDOS_control_ *–* tDOS_passivated_)/ tDOS_control_) for these materials to compare their passivations capabilities, as shown in Figure 4d. It is shown that PEAI can reduce the density of trap band I most efficiently among these four materials, which is consistent with the trend of the efficiency change for these devices (Figure S23, Supporting Information). Meanwhile, the selection of the halide ion I^−^ or Br^−^ does not change the *η* significantly, indicating that the passivations capability of Br^−^ is similar to I^−^ on the surface defects that cause trap band I in FAPbI_3_ perovskite films. Interestingly, it is found that PEAI (PEABr) owns a higher *η* than OAI (OABr), especially in deeper areas from the surface of the perovskite films. We believe that this difference should be ascribed to the difference between PEA^+^ and OA^+^ cations. Previous works have revealed that positively charged cations like PEA^+^ and OA^+^ could passivate the negatively charged surface defects such as undercoordinated I^−^, anti‐site ${\mathrm{PbI}}_{3}^{-}$ and cation vacancies through ionic bonding or hydrogen bonding.^[^ ^45^, ^46^, ^47^, ^48^, ^49^ ^]^ Considering the *E*_d_ of trap band I (0.14–0.18 eV), we argue that the undercoordinated I^−^ could be the chemical origin for trap band I,^[^ ^50^, ^51^ ^]^ which is consistent with previous results.^[^ ^16^ ^]^ Given the relatively smaller size of PEA^+^ compared to OA^+^, the PEA^+^ ions are more likely to diffuse into the bulk of the perovskite film through the pathway like grain boundaries and therefore passivate the undercoordinated I^−^ defects there^[^ ^52^ ^]^ (as schematically illustrated in Figure 4e). This leads to a higher *η* for PEA^+^ ions compared to OA^+^ in the sub‐surface areas of the perovskite films.

## 3 Conclusion

In conclusion, we investigated the RC coupling effect on the capacitance spectroscopy results of n*–*i*–*p structured perovskite solar cells and discovered that an enhanced RC coupling effect caused by the carrier freeze‐out of the HTL resulted in an RC TAS peak resembling a defect‐induced trap state TAS peak in PSCs. The RC coupling and defect‐induced trap state TAS peaks can be distinguished by introducing an additional *R*_s_ during the TAS measurement. More importantly, we proposed an effective method to calibrate the measured TAS results for PSCs, which generates the actual density and spatial distribution of defect‐induced trap states in perovskite films, making the TAS method further appliable for the study of the defect landscapes in perovskite films.

## Conflict of Interest

The authors declare no conflict of interest.

## Supporting information

Supporting Information

## Data Availability

The data that support the findings of this study are available from the corresponding author upon reasonable request.
