# Supplementary material for: Eliminating Resistance–Capacitance Coupling Shielding for Depicting the Defect Landscape in Perovskite Solar Cells by Capacitance Spectroscopy
Source: Adv Sci (Weinh). 2024 Jun 19;11(31):2403984. doi: 10.1002/advs.202403984 (PMC11336947; doi:10.1002/advs.202403984)
Supplement: Supplementary file 1 — Supporting Information [file ADVS-11-2403984-s001.docx]

Supporting Information for

**Eliminating resistance-capacitance coupling shielding for depicting the defect landscape in perovskite solar cells by capacitance spectroscopy**

Biao Li^‡1^, Daoyong, Zhang^‡1^, Zhengyi, Ni^1^***,** Pengjie Hang^1^, Yuxin Yao^1^, Chenxia Kan^1^, Xuegong Yu^1,2^*****, Deren Yang^1,2^

^1^State Key Laboratory of Silicon and Advanced Semiconductor Materials and School of Materials Science and Engineering, Zhejiang University, Hangzhou, 310027, China

^2^ZJU-Hangzhou Global Scientific and Technological Innovation Center, Hangzhou, 311200, China

^‡^These authors contributed equally.

*****Corresponding author

E-mail: zyni@zju.edu.cn; yuxuegong@zju.edu.cn

This Supporting Information includes:

Experimental Methods

Figures S1 to S28

Tables S1-S5

Notes S1-S4

**Experimental Methods**

**Materials**

All the materials were purchased from Sigma-Aldrich unless the following material. FAI, PbI_2_, MABr, MACl, CsI, Spiro and BCP were purchased from Xi’an Polymer Light Technology Corp. PTAA was purchased from Luminescence Technology Corp. SnO_2_ H_2_O colloidal dispersion liquid was purchased from Alfa Aesar. Acetone, isopropanol and ethyl alcohol (for cleaning) was purchased from Sinopharm Chemical Reagent Co., Ltd. ITO substrates were purchased from Hangzhou Hong Shi Technology Corp.

**Perovskite solar cells fabrication**

The ITO glasses were sequentially cleaned in deionized water, ethyl alcohol, acetone and isopropanol by sonication for 5 min. Before deposition of electron transporting layer, all ITO glasses were further cleaned for 20 min by UV-ozone machine. To deposit electron transporting layer, SnO_2_ precursor solution was prepared by mixing 15% SnO_2_ water colloidal dispersion liquid with deionized water with a volume ratio of 1:5. The precursor solution was spin coated onto ITO glasses at 3000 rpm for 30 s. SnO_2_ layer was annealed under ambient air at 150 ℃ for 1 h. For perovskite films fabrication, 461 mg of PbI_2_ was dissolved in 900 μL N,N-dimethylformamide (DMF) and 100 μL dimethyl sulfoxide (DMSO). 50 μL precursor solution was spin-coated onto SnO_2_ at 1500 rpm for 30 s and then heated at 70°C for 1 minute. Subsequently, a solution comprising of FAI (90 mg) and MACl (9 mg) in 1 ml of isopropanol and then 70 µl of the resulted solution was spin-coated onto the PbI_2_ films at a spinning rate of 1750 rpm for 30 s. The perovskite films were removed from a nitrogen-filled glove box and transferred to the ambient air for annealing at 150°C for 15 minutes. For Spiro solution, 17.5 μL Li-TFSI acetonitrile solution (520 mg mL^-1^) and 28.75 μL TBP were added into 72.25 mg mL^-1^ Spiro chlorobenzene solution. PTAA HTL was prepared in chlorobenzene solution (30 mg mL^-1^), doped with 11% TPFB. The doped PTAA solution was stirred at 45°C overnight to ensure full dissolving and doping of PTAA. The HTL solution was spin coated onto perovskite films at 3000 rpm for 30 s.

P3HT HTL was prepared by spin coating 10 mg mL^-1^ P3HT chlorobenzene solution at 3000 rpm for 30 s onto the perovskite films. Finally, 100 nm Au was deposited on the top of the HTL under a vacuum of less than 5×10^-4^ Pa by thermal evaporation system.

The fabrication of p-i-n PSCs: The ITO substrates were sequentially cleaned with deionized water, acetone, isopropanol and ethanol by ultrasonication for 5 min in each solvent. The cleaned ITO substrates were then dried in air oven and treated with UV ozone for 20 min before use. The hole transport layer (HTL) was fabricated by using 2PACZ. A 1 mg mL^-1^ 2PACz solution in ethanol was spin-coated on ITO in a nitrogen glove box at 3000 rpm for 30 s and annealed at 100°C for 10 minutes. The 1.4 M perovskite precursor solution was prepared in DMF: DMSO (4:1/v:v) solvent according to a formula of Cs_0.05_(FA_0.95_MA_0.05_)_0.95_Pb(I_0.95_Br_0.05_)_3_ with 3% excess amount of PbI_2_, and 15 mol% of MACl. The perovskite solutions were spin-coated at 1000 rpm for 10 s and 4000 rpm for 40 s. At the last 8 s of the second step, 150 μL CB was dropped as antisolvent. The films were then annealed at 100°C for 15 min. A 2 mg mL^-1^ solution of PEABr in IPA was spin-coated at 5000 rpm for 30 s and annealed for 5 min at 100°C. Afterwards, 20 nm C60 at a rate of 0.2 Å s^-1^, 5 nm BCP at a rate of 0.2 Å s^-1^ and 100 nm silver electrode at a rate of 1.0 Å s^-1^ were thermally evaporated, respectively.

**Film and device characterization**

The current density–voltage (*J*–*V*) curves were determined using a Keithley 2400 source measurement unit under a simulated AM1.5G spectrum with a Newport 94022A solar simulator. The light intensity was calibrated by a standard Si reference solar cell (PVM937, Newport) with KG5 filter calibrated by Newport Corporation TAC-PV Lab. The spectral mismatch correction factor is *M* = 0.994 ± 0.001. The *J*–*V* curves of devices were measured both in reverse scan (1.2V to 0 V with a step of 0.013 V and a dwelling time of 10 ms at each step) and forward scan (0 V to 1.2 V with a step of 0.013 V and a dwelling time of 10 ms at each step). The device was tested at room temperature (25 ± 5 ℃) with a relative humidity of 30 ± 10% under ambient conditions. All devices were measured without pre-conditioning such as light-soaking the device. The device was tested with a calibrated mask with an aperture area of 0.0528 cm^2^. Capacitance, TAS and TID tests were performed by a LCR meter (Keysight E4980A). For the *C* – *V* test, it was performed with an ac voltage of 20 mV and the ac frequency was 10 KHz. For the TAS test, it was performed with a dc voltage of 0 V (unless otherwise specified) and an ac voltage of 20 mV. The frequency scan was from 20 Hz to 1 MHz. For the TID test, it was performed with a dc voltage of 0.8 V and an ac voltage of 20 mV with a frequency of 10 KHz. EBIC data was acquired through HITACH S-3400N, Gatan DIGISCAN II and low-noise current preamplifier (SRS Model SR570).

**
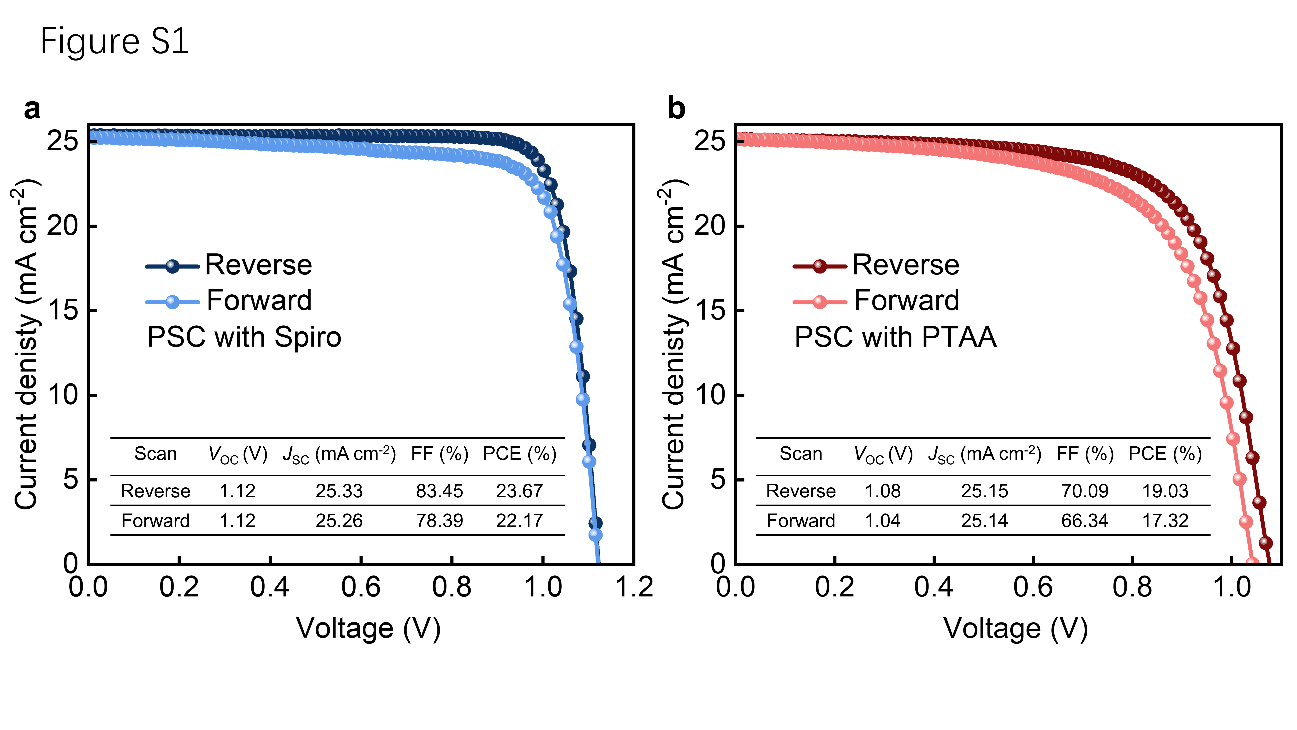
**

**Figure S1.** *J-V* curves for representative PSCs with different HTLs: (a) Spiro; (b) PTAA.


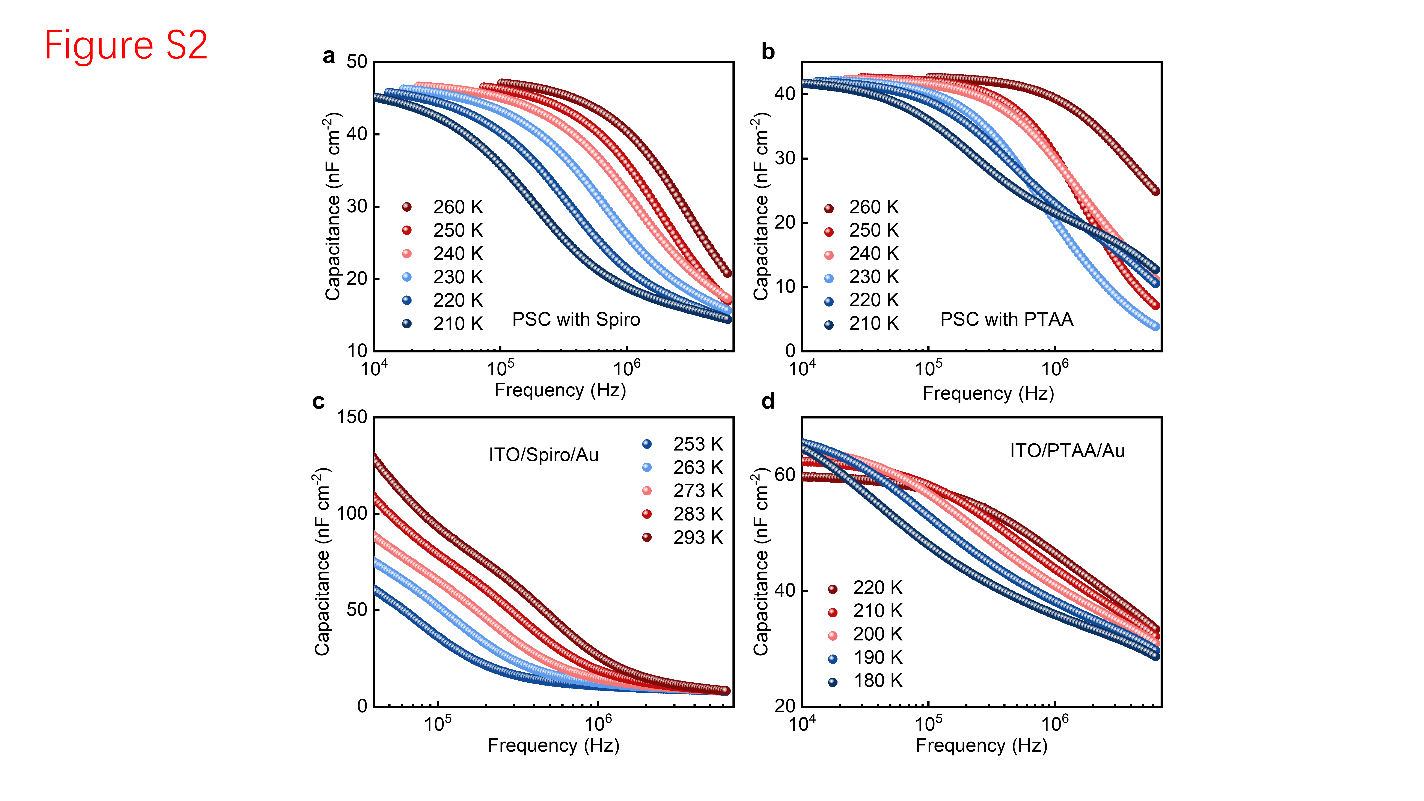


**Figure S2.** Raw capacitance data for devices with different configurations with a temperature step of 10 K: (a) a PSC with Spiro, (b) a PSC with PTAA, (c) a Spiro only device, (d) a PTAA only device.


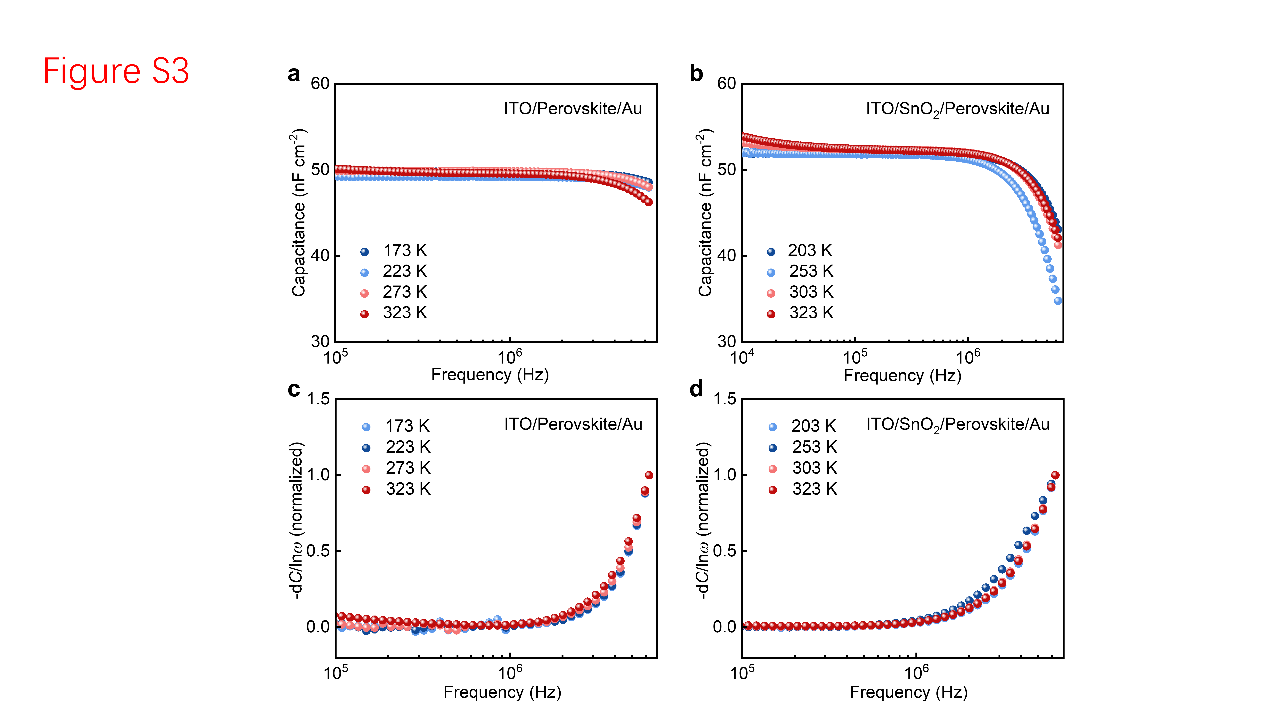


**Figure S3.** Raw capacitance data for different devices: (a) ITO/Perovskite/Au; (b) ITO/SnO_2_/Perovskite/Au. TAS results for different devices: (c) ITO/Perovskite/Au; (d) ITO/SnO_2_/Perovskite/Au.

**
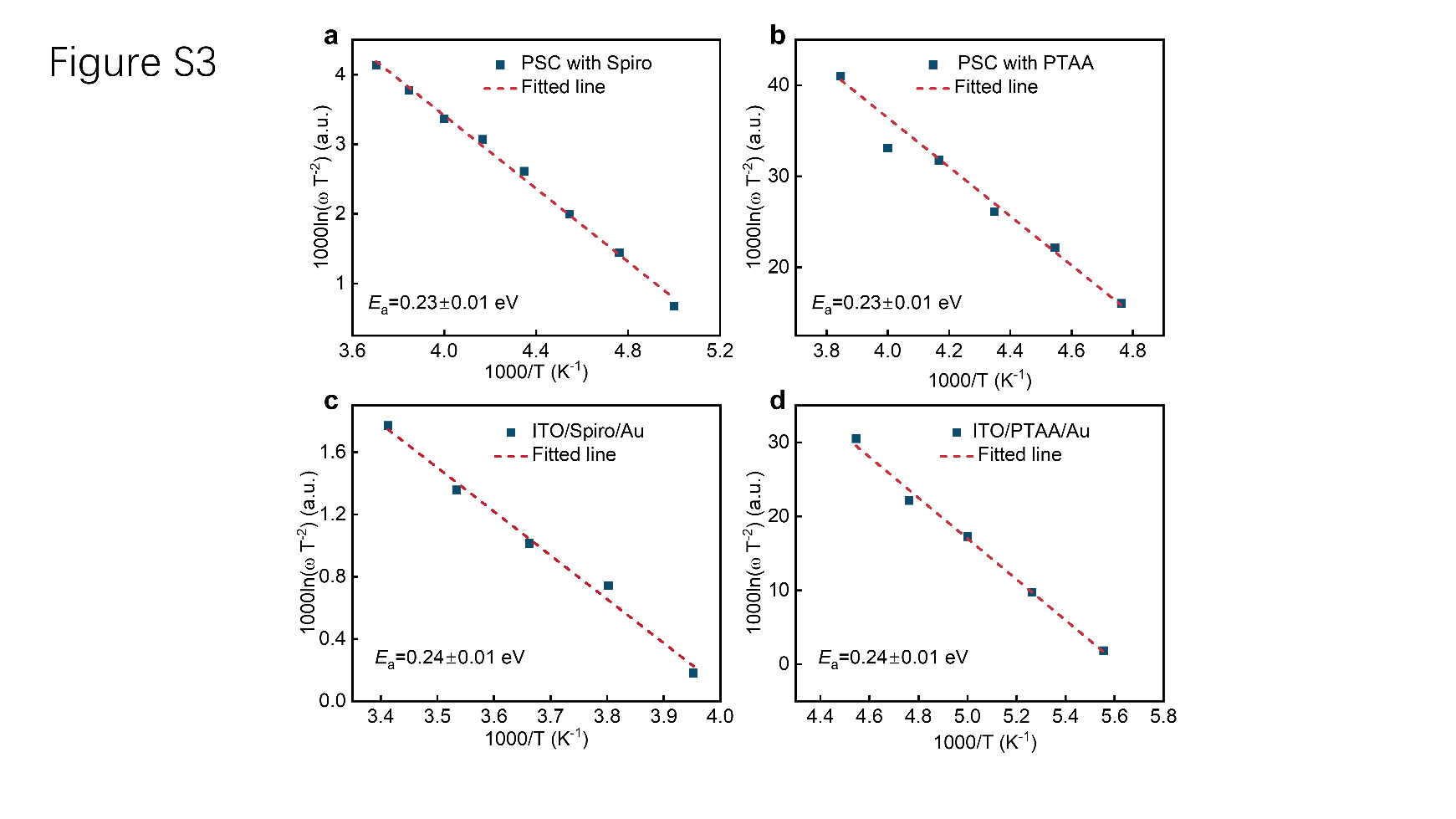
**

**Figure S4.** Arrhenius plots of the capacitance signature in the PSCs with HTLs: (a) Spiro; (b) PTAA. Arrhenius plots of the capacitance signature in the HTL only devices: (c) Spiro; (d) PTAA.

**
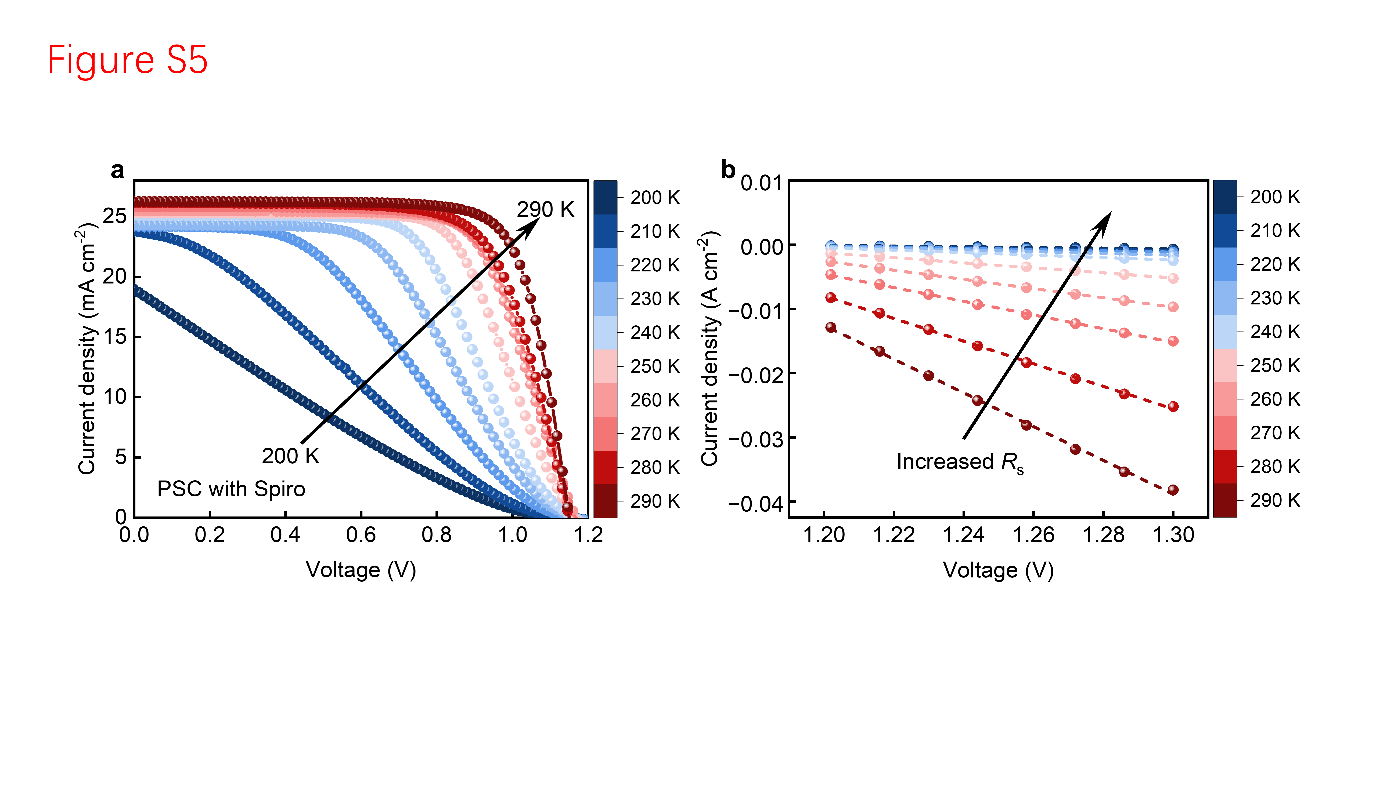
**

**Figure S5.** (a) *J-V* curves of a PSC with Spiro at different temperatures. (b) extraction of *R*_s_ using the *J-V* curves (1.2 – 1.3 V) of a PSC with Spiro at different temperatures.

**Table S1.** *J*-*V* parameters of a PSC at different temperatures.

| Temperature (K) | *V*_OC_ (V) | *J*_SC_ (mA cm^-2^) | FF (%) | PCE (%) | *R*_s_ (Ω cm^2^) |
| --- | --- | --- | --- | --- | --- |
| 200 | 1.14 | 18.96 | 20.10 | 4.33 | 193.03 |
| 210 | 1.14 | 23.77 | 25.83 | 6.99 | 138.01 |
| 220 | 1.16 | 24.19 | 39.10 | 10.94 | 103.86 |
| 230 | 1.16 | 24.29 | 51.04 | 14.42 | 74.08 |
| 240 | 1.16 | 25.09 | 58.67 | 17.13 | 51.81 |
| 250 | 1.17 | 25.30 | 66.06 | 19.49 | 25.43 |
| 260 | 1.17 | 25.48 | 70.21 | 20.88 | 13.92 |
| 270 | 1.16 | 25.82 | 70.32 | 21.03 | 9.38 |
| 280 | 1.15 | 26.15 | 70.86 | 21.31 | 5.67 |
| 290 | 1.15 | 26.24 | 76.84 | 23.18 | 3.80 |


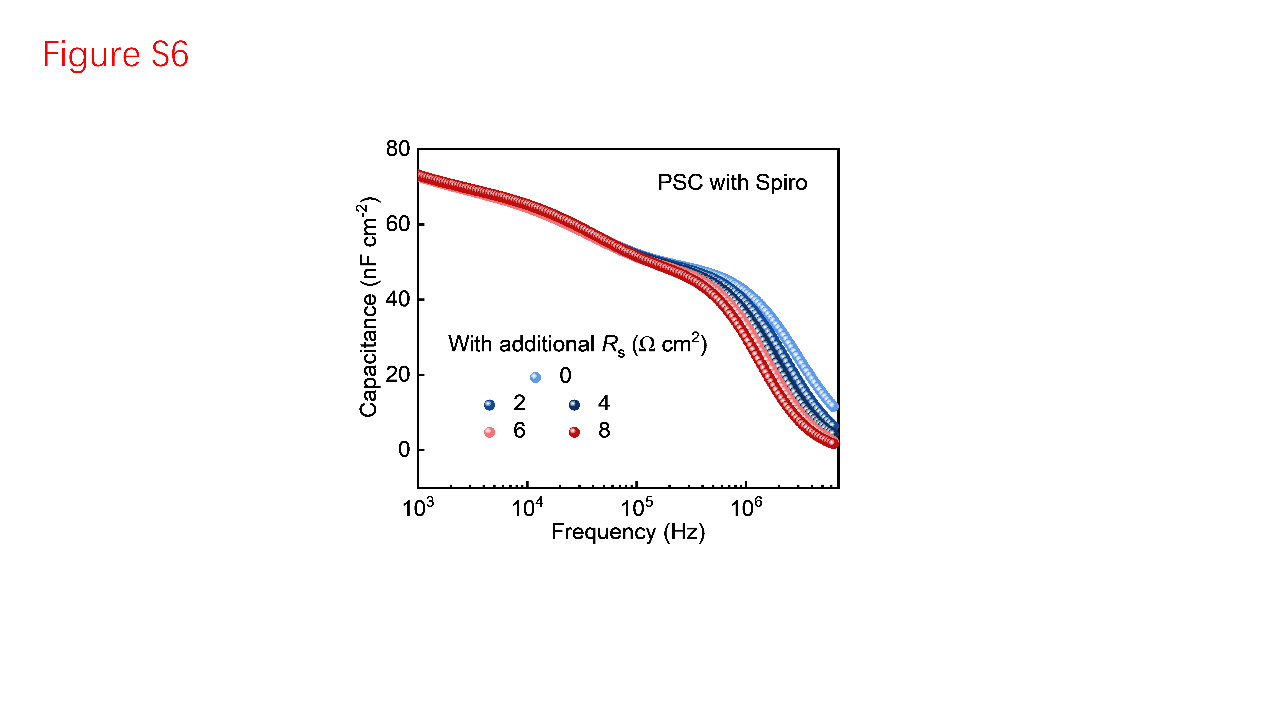


**Figure S6.** Raw capacitance data for Fig. 2b.

**
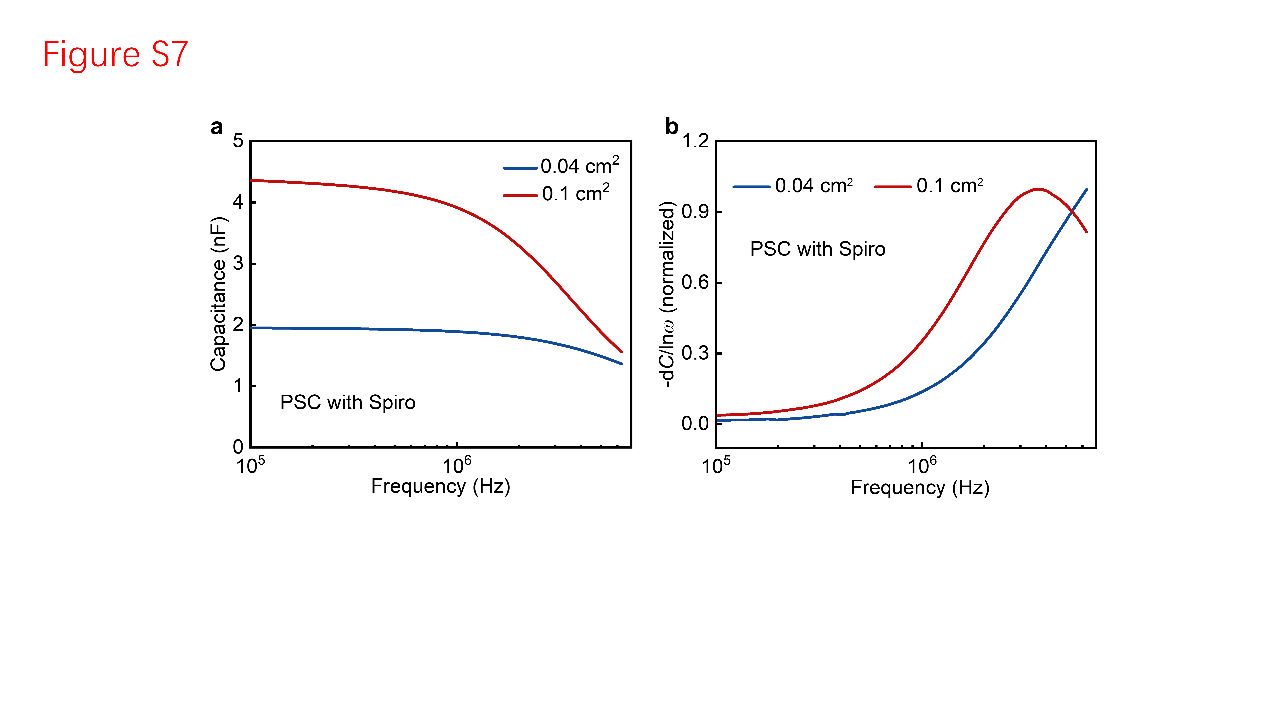
**

**Figure S7.** Raw capacitance data (a) and TAS results (b) for PSCs with Spiro and different active area.

**
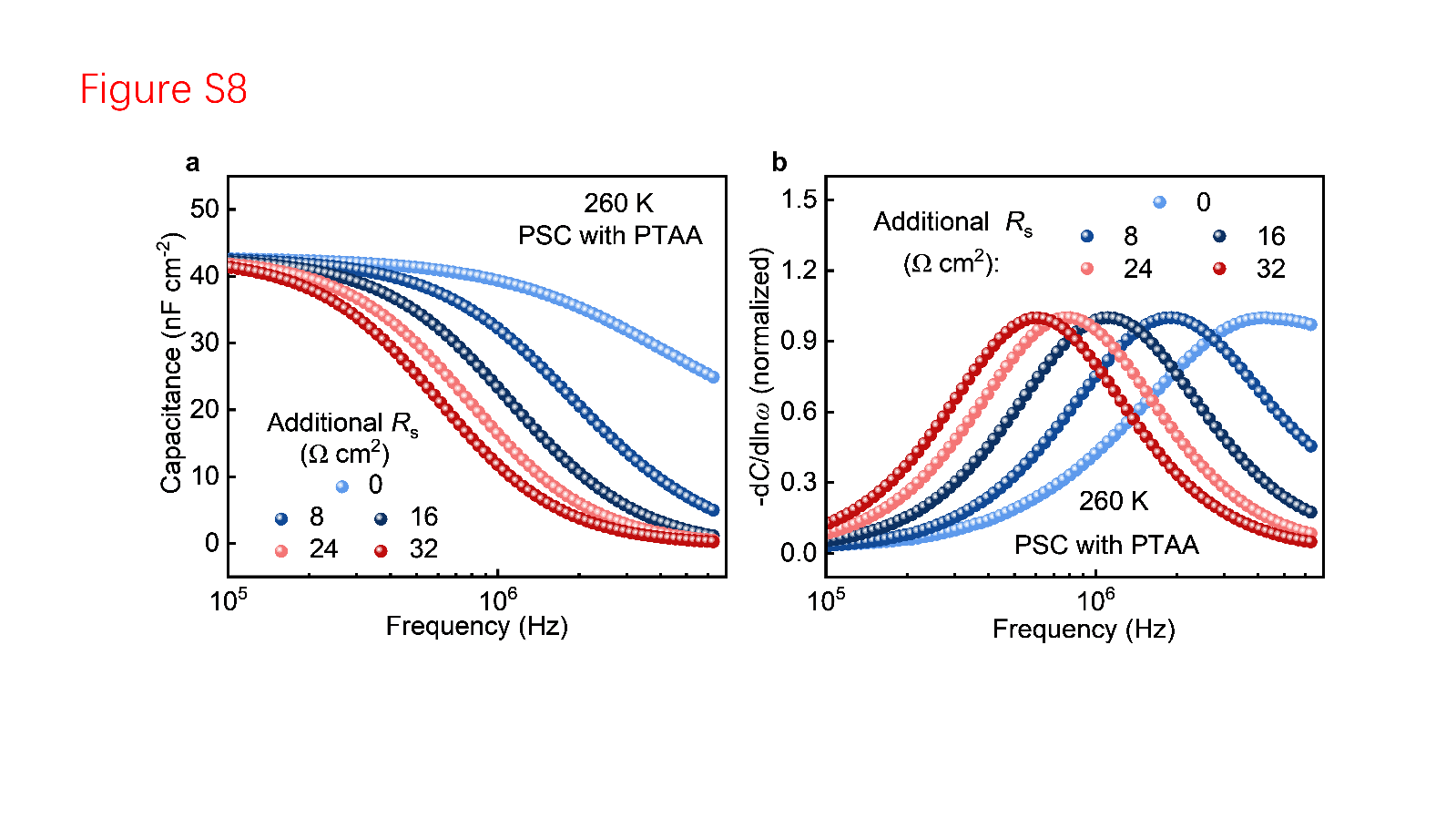
**

**Figure S8.** Raw capacitance data (a) and TAS results (b) for a PTAA PSC with increased additional series resistance at 260 K.


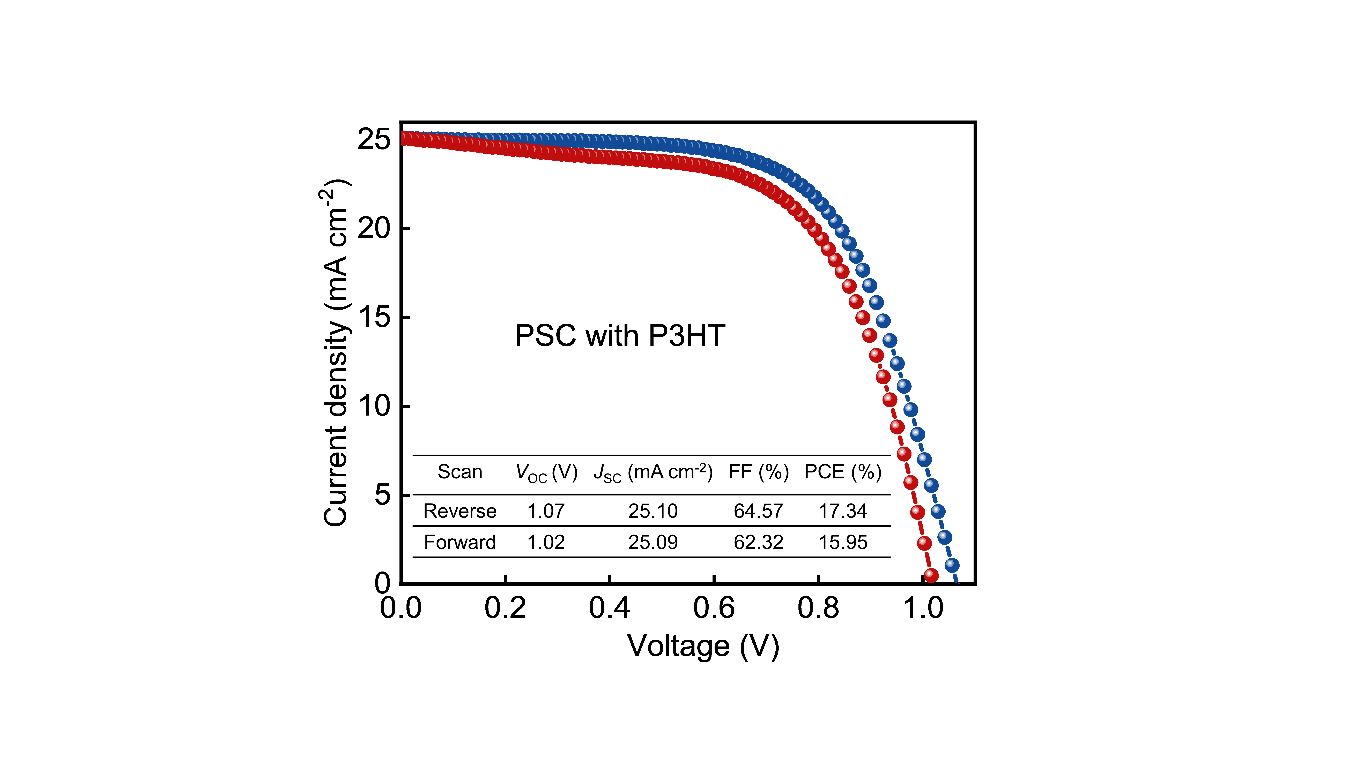


**Figure S9.** *J* - *V* curves of the PSC with P3HT.


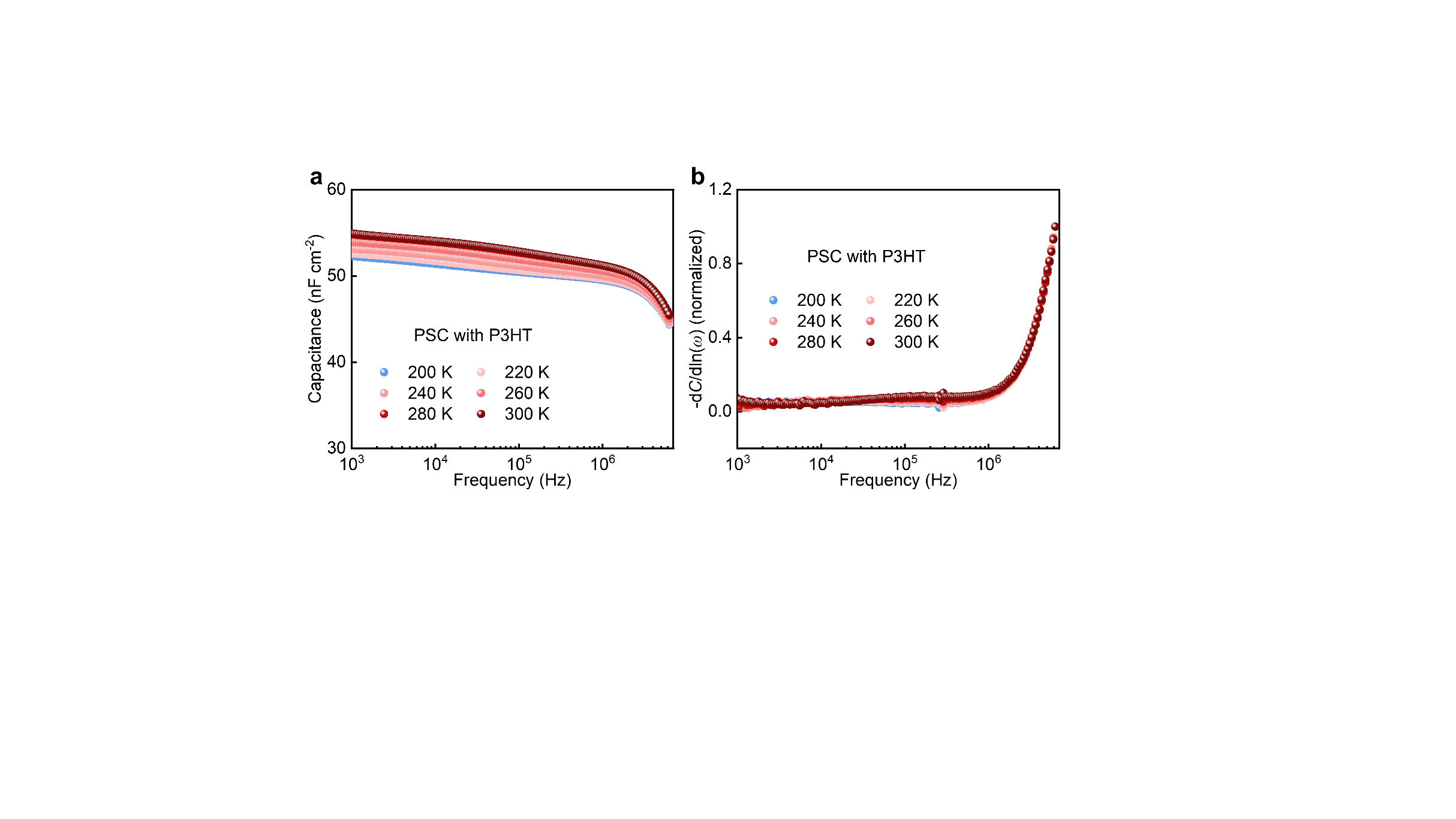


**Figure S10.** a, *C* – *ω* plot of the fabricated perovskite solar cells with P3HT. b, d*C*/dln*ω* – *ω* plot of the fabricated device.

**Note S1 Drift-diffusion simulations**

We performed drift-diffusion simulations via SCAPS-1D software developed by the group of Prof. Burgelman at the University of Gent to simulate the TAS of PSCs^1^. This software solves the continuity equation for electrons and holes together with the Poisson equation. In the simulation, we construct a n-i-p PSC. Parameters used in our simulations are listed in Table S2-S4. It is widely reported that mobile ions can influence the capacitance results of perovskite solar cells. However, the drift-diffusion simulations via SCAPS-1D software do not include mobile ions into calculations. We note that the mobile ions are not a concern in our study because our simulations focus on the high frequency (> 10^3^ Hz) region, while mobile ions only introduce a capacitance step into the TAS results obtained at frequencies lower than 10^3^ Hz due to their limited mobilities^2,3^. The excellent agreement between the simulation results and the experimental results demonstrates the validation of our SCAPS simulations.

**Table S2.** Perovskite parameters used for simulations^4^.

| Parameter | Symbol | Value | Unit |
| --- | --- | --- | --- |
| Thickness | *d*_pero_ | 800 | nm |
| Energy bandgap | *E*_g_ | 1.52 | eV |
| Conduction band minimum | *E*_c_ | -3.9 | eV |
| Relative dielectric constant | *ε_r_* | 30 | / |
| Effective conduction band density of states | *N*_C_ | 1 × 10^19^ | cm^-3^ |
| Effective valence band density of states | *N*_V_ | 1 × 10^19^ | cm^-3^ |
| Electron mobility | *μ_e_* | 2 | cm^2^ V^-1^ s^-1^ |
| Hole mobility | *μ_h_* | 2 | cm^2^ V^-1^ s^-1^ |
| Effective doping density | *N*_A_ | 2 × 10^16^ | cm^-3^ |
| Temperature | *T* | 300 | K |
| Defect energy level (above *E*_v_) | *E*_d_ | 0.48 | eV |
| Defect density | *N*_T_ | 8 × 10^15^ | cm^-3^ |
| Defect capture cross section | *σ* | 1 × 10^-15^ | cm^2^ |

**Table S3.** SnO_2_ parameters used for simulations^5^.

| Parameter | Symbol | Value | Unit |
| --- | --- | --- | --- |
| Thickness | *d* | 20 | nm |
| Energy bandgap | *E*_g_ | 3.2 | eV |
| Conduction band minimum | *E*_c_ | -4.0 | eV |
| Relative dielectric constant | *ε_r_* | 9 | / |
| Effective conduction band density of states | *N*_C_ | 2.2 × 10^18^ | cm^-3^ |
| Effective valence band density of states | *N*_V_ | 1.8 × 10^19^ | cm^-3^ |
| Electron mobility | *μ_e_* | 10 | cm^2^ V^-1^ s^-1^ |
| Hole mobility | *μ_h_* | 0.26 | cm^2^ V^-1^ s^-1^ |
| Effective doping density | *N*_D_ | 1 × 10^18^ | cm^-3^ |

**Table S4.** Spiro parameters used for simulations^4^.

| Parameter | Symbol | Value | Unit |
| --- | --- | --- | --- |
| Thickness | *d* | 20 | nm |
| Energy bandgap | *E*_g_ | 3 | eV |
| Conduction band minimum | *E*_c_ | -2.42 | eV |
| Relative dielectric constant | *ε_r_* | 3 | / |
| Effective conduction band density of states | *N*_C_ | 1 × 10^19^ | cm^-3^ |
| Effective valence band density of states | *N*_V_ | 1 × 10^19^ | cm^-3^ |
| Electron mobility | *μ_e_* | 2 × 10^-4^ | cm^2^ V^-1^ s^-1^ |
| Hole mobility | *μ_h_* | 2 × 10^-4^ | cm^2^ V^-1^ s^-1^ |
| Effective doping density | *N*_A_ | 2 × 10^18^ | cm^-3^ |


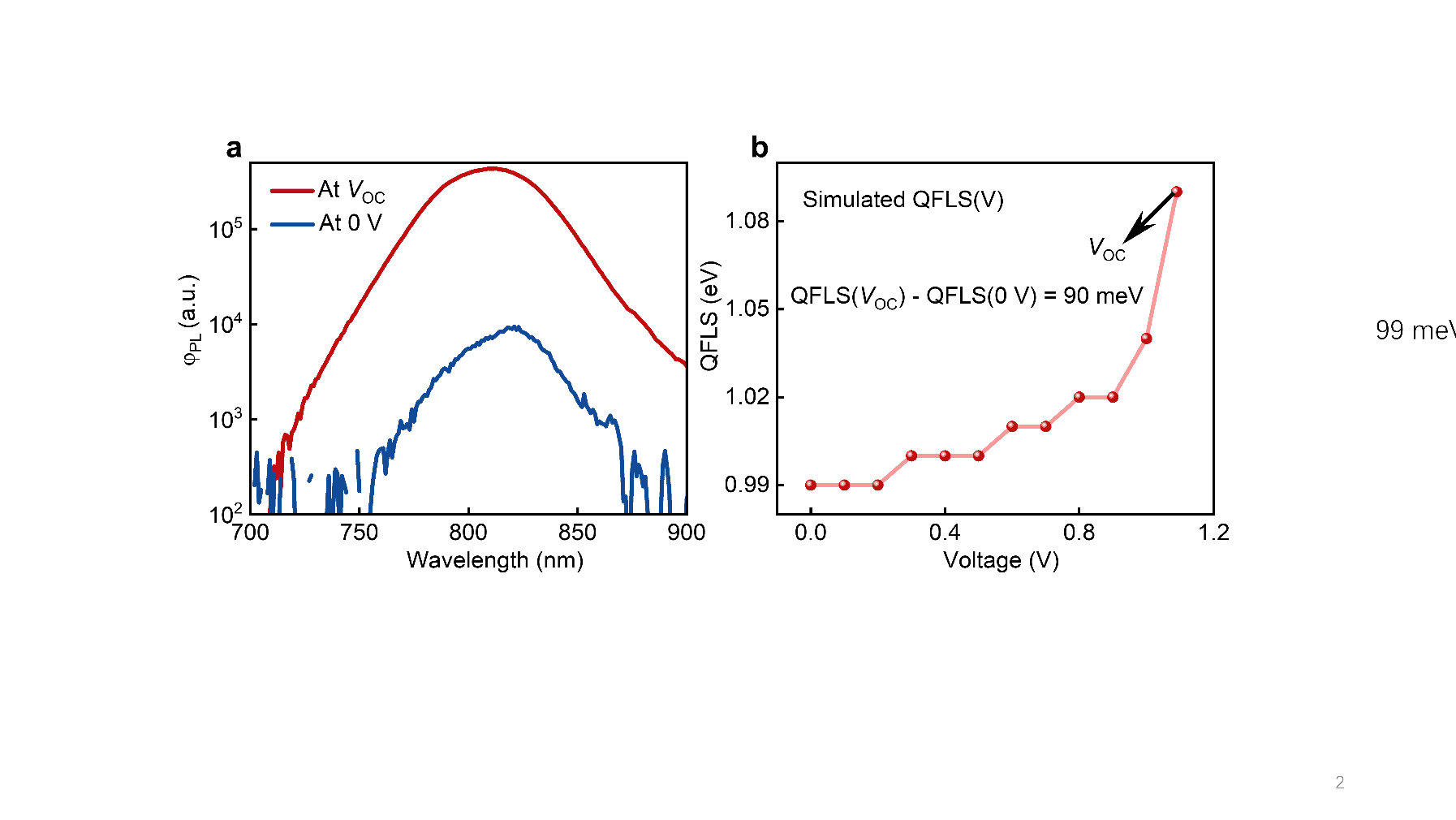


**Figure S11.** a, Steady state PL results at 0 V and *V*_OC_. b, Simulated QFLS(V) plot produced by SCAPS.

**
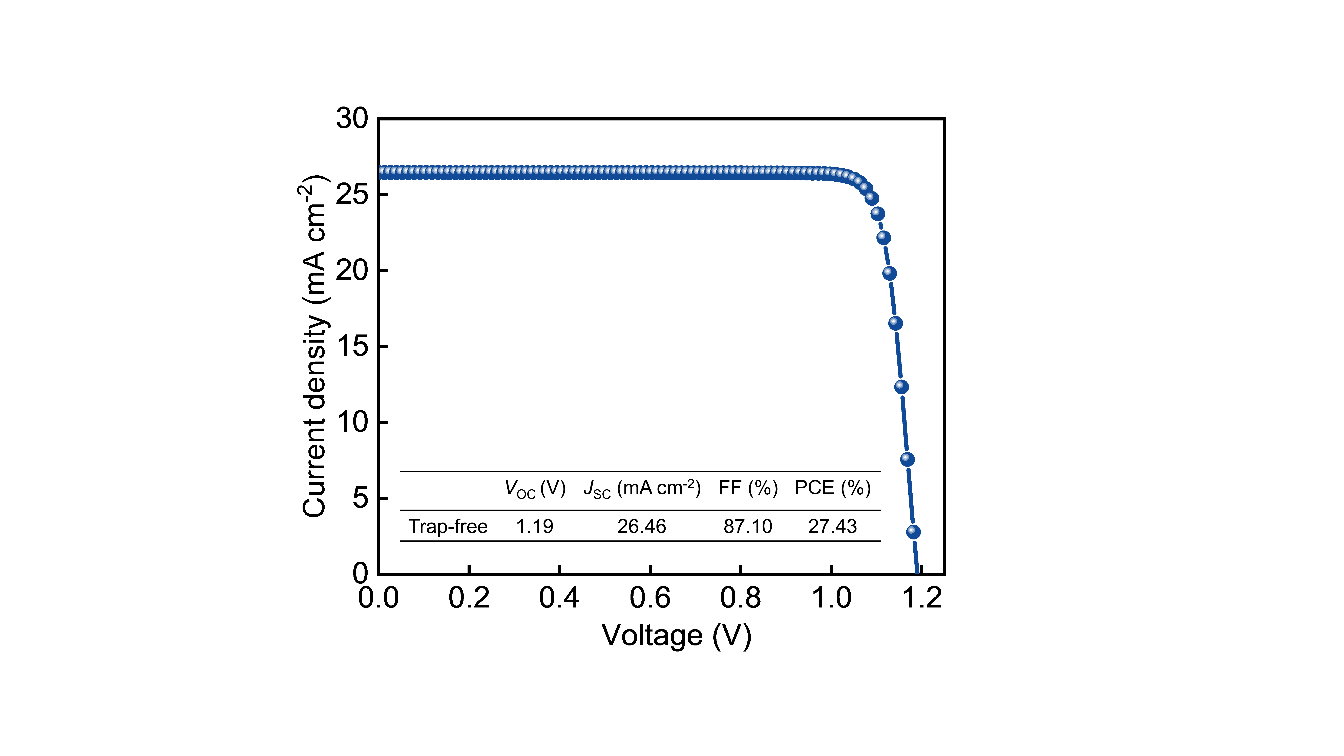
**

**Figure S12.** Simulated *J* - *V* curves of the modelled trap-free perovskite solar cell.


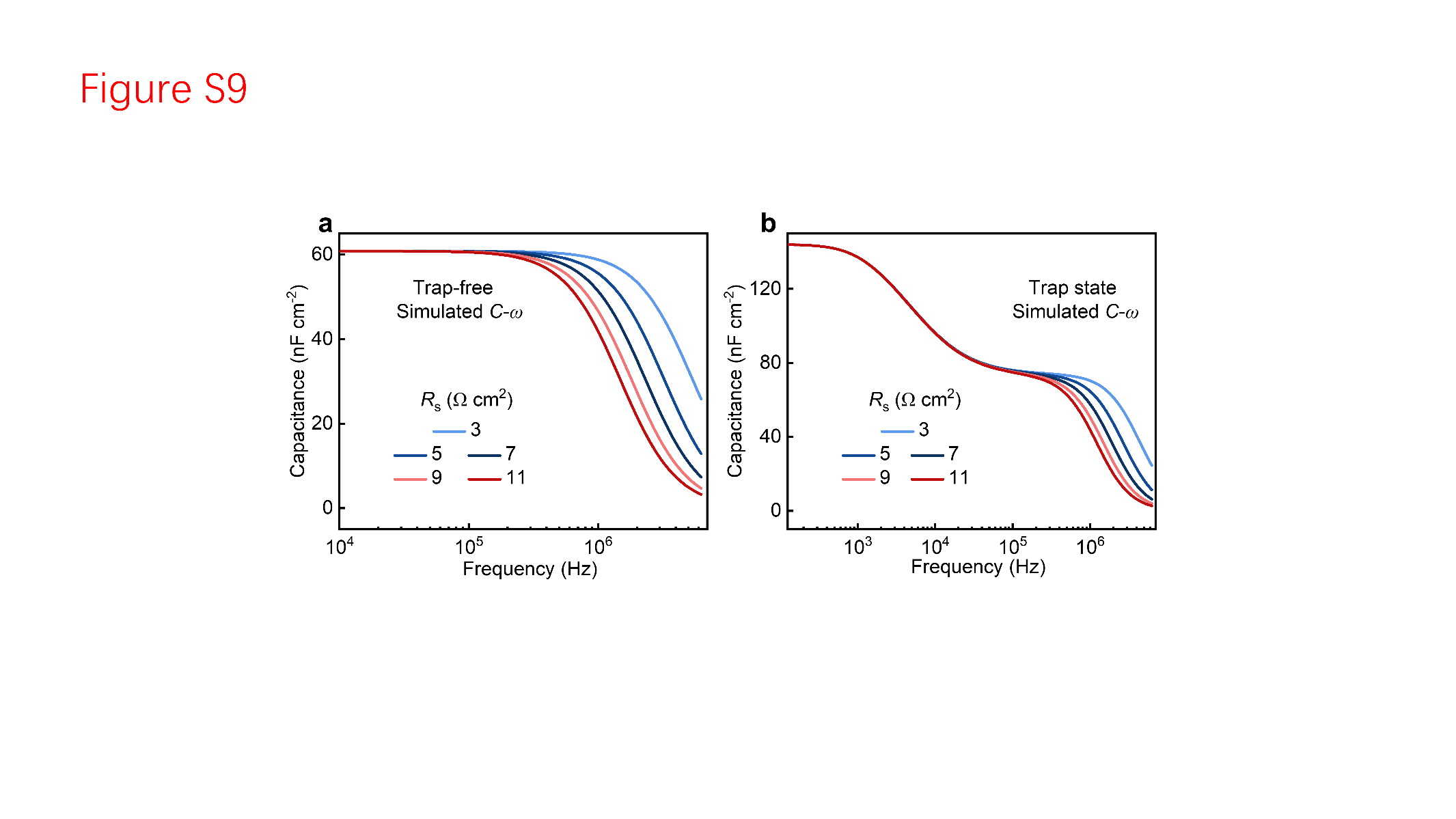


**Figure S13.** (a) Raw capacitance data for Fig. 2c; (b) Raw capacitance data for Figure 2d.


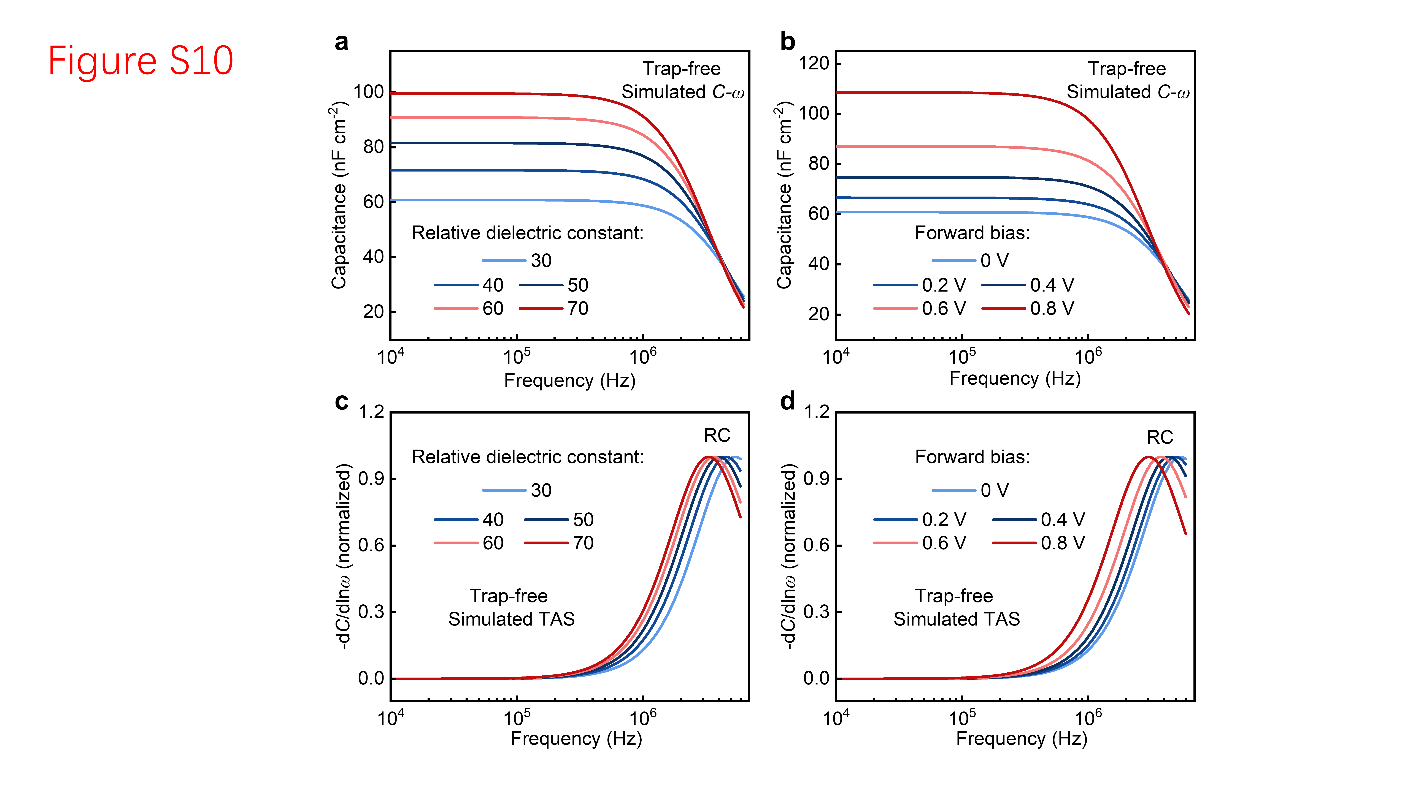


**Figure S14.** Raw capacitance data for a n-i-p trap-free PSC: (a) with different relative dielectric constants; (b) with different forward biases. Normalized simulated TAS results for a n-i-p trap-free PSC: (c) with different relative dielectric constants; (d) with different forward biases.

**Note S2 Capacitance induced by trap states**

In the main text, we use Equation (2) to describe the frequency-dependent *C*_T_ of trap states. We here mentioned that this Equation (2) is derived based on Cole-Cole equation with a single response mode^6^. This means that this equation is only applicable to the point defect in perovskites, which has a single energy level in the energy band gap of perovskites. Therefore, this equation is not applicable to the extended defects or band tail states which have continuous energy levels in the energy band gap of perovskites. For extended defects, up to now, there are no literatures reporting the existence of extended defects via TAS tests. For tail states, their attempt-to-escape frequency derived by their shallow characteristic energy depth (tens of meV) is much faster than the attempt-to-escape frequency of measured trap states (10^5^ – 10^6^ Hz). Therefore, we choose Equation (2) to calibrate the TAS results at the frequency domain of 10^5^ – 10^6^ Hz.

**Note S3 TAS tests**

According to the theory of TAS^7,8^, the frequency at the peak (*ω_0_*) is related to emission rates of a trap state as described in

| $\omega_{0}={2v}_{0}exp(-\frac{E_{d}}{k_{B}T})$ | (S1) |
| --- | --- |

where *v_0_* is the attempt-to-escape frequency, *T* is temperature, *k*_B_ is Boltzmann constant. *E_d_* is the energy depth of a trap state. We note that *v*_0_ can be described by *ξ*_0_T^2^, where *ξ*_0_ is a temperature independent constant^7^. Based on Equation (S1), an Arrhenius equation can be derived as described in

| $\ln\left( \frac{\omega_{0}}{T^{2}} \right)={ln(2\xi}_{0})-\frac{E_{d}}{k_{B}T}$ | (S2) |
| --- | --- |

Therefore, we can calculate *v*_0_ based on the intercept of the linear fitting line of the ln(*ω*_0_/*T*^2^) – 1/*T* plot. Based on Equation (S1), a demarcation energy (*E_ω_*) can be defined

| $E_{\omega}=k_{B}Tln(\frac{{2v}_{0}}{\omega})$ | (S3) |
| --- | --- |

In addition, the density states of the trap state (tDOS) are described by

| $\mathrm{tDOS}=-\frac{V_{bi}}{Aqk_{B}Td}\frac{dC}{dln(\omega)}$ | (S4) |
| --- | --- |

where *A* is the device area, *d* is the thickness of perovskites which is ~1 μm for the fabricated films, *V*_bi_ is the built-in potential of the device. We calculated the doping density (*N*) of perovskite films, which is 1.31 × 10^16^ cm^-3^ for the fabricated perovskite films which is higher than the resolution limit of 2 × 10^15^ cm^−3^ for a 1000-nm-thick perovskite layer, which suggests that the depletion model can be applied to our fabricated device^2,9-10^. However, it is found the *C* – *V* curve (Figure S14) is influenced by ion migration in perovskite films as indicated by the onset of the Mott-Schottky plot^2^. Directly utilizing the intercept point to extract the *V*_bi_ will lead to a substantially overestimation, since the mobile ions can diffuse back to the bulk of perovskite films and change the net space charge density of perovskite films at this bias region^2^. Therefore, we need to calculate the apparent doping density (*N*_app_) of perovskite films at 0 V which is compensated by mobile ions in perovskite films. We performed transient ion drift (TID) tests to derive the density of mobile ions (*N*_ion_), which is a verified method in studying the ion migration process in perovskite solar cells^11,12^. The capacitance transient is shown in Figure S15. The *N*_ion_ can be calculated according to^11^

| $N_{\mathrm{ion}}\cong2N\left( \frac{{\Delta C}_{0}}{C_{\infty}} \right)$ | (S5) |
| --- | --- |

where *C*_∞_ is capacitance of equilibrium at the applied voltage, ∆*C*_0_ is the difference between *C*_∞_ and the capacitance at time zero. Then, we can calculate the *N*_app_ by subtracting *N*_ion_ from *N*. We then calculated the *V*_bi_ of perovskite solar cells at 0 V based on the thickness and the *N*_app_ of perovskite films and the calculated *V*_bi_ is 1.3 V.

We perform the same tr-PL vs. fluence measurement (Figure S17) on the fabricated device to further validate the carrier density of perovskite films^13^. We extract the initial amplitude of the tr-PL curves at each light fluence and calculate the induced carrier density based on the light fluence. Figure S17 shows that, as far as the FAPbI_3_ perovskite adopted in this work is concerned, the initial tr-PL amplitude depends linearly on the induced carrier density, and consequently, the doping is larger than the highest induced carrier density of 2.19 × 10^16^ cm^-3^, which is consistent with the carrier density derived from the *C* - *V* tests.

**
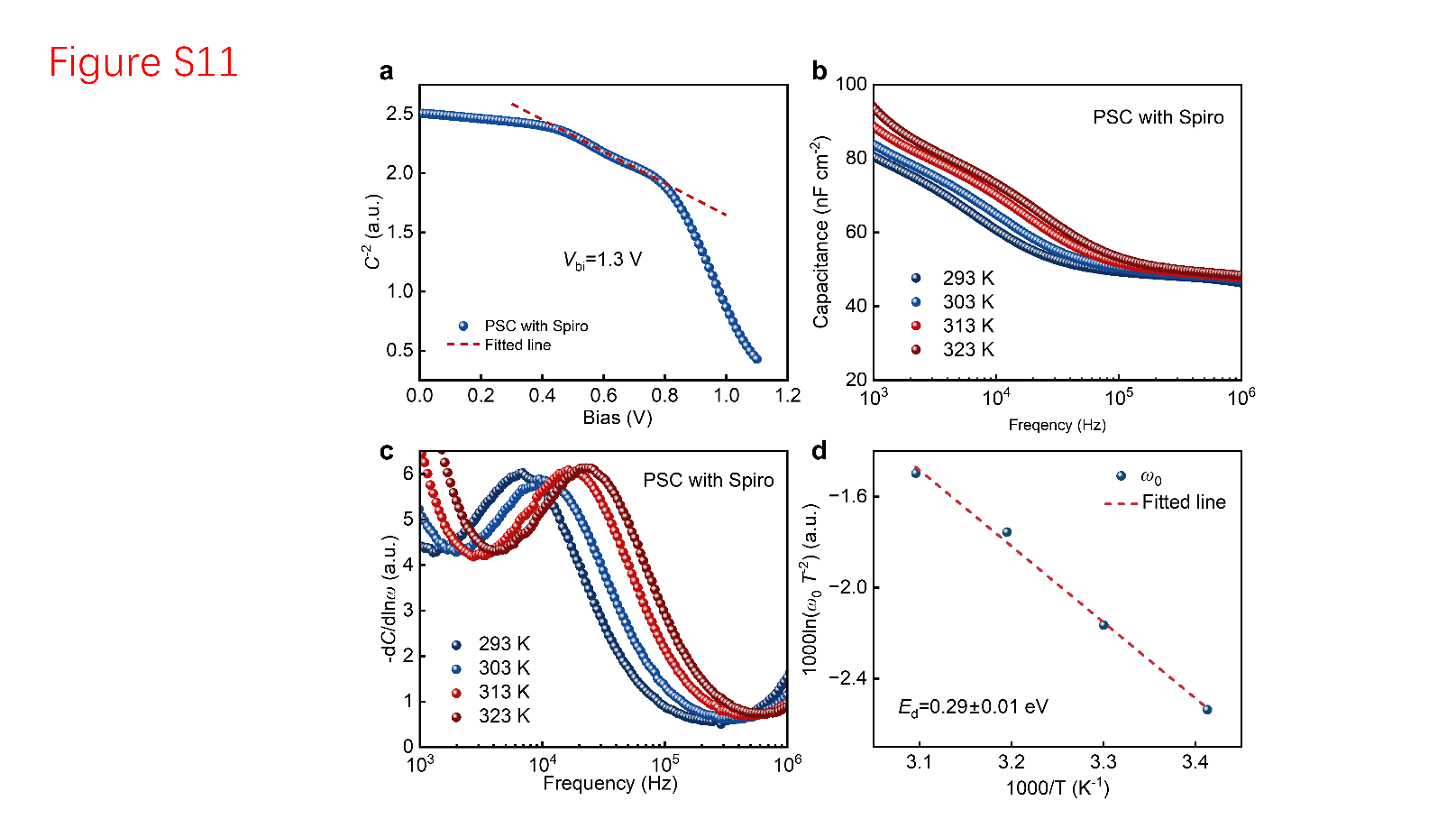
**

**Figure S15.** (a) Mott-Schottky results for a PSC with Spiro. (b) Raw capacitance data for a PSC with Spiro at different temperatures. (c) Temperature dependent TAS tests for a PSC with Spiro at different temperatures. (d) Arrhenius plots of the capacitance signature for a PSC with Spiro.


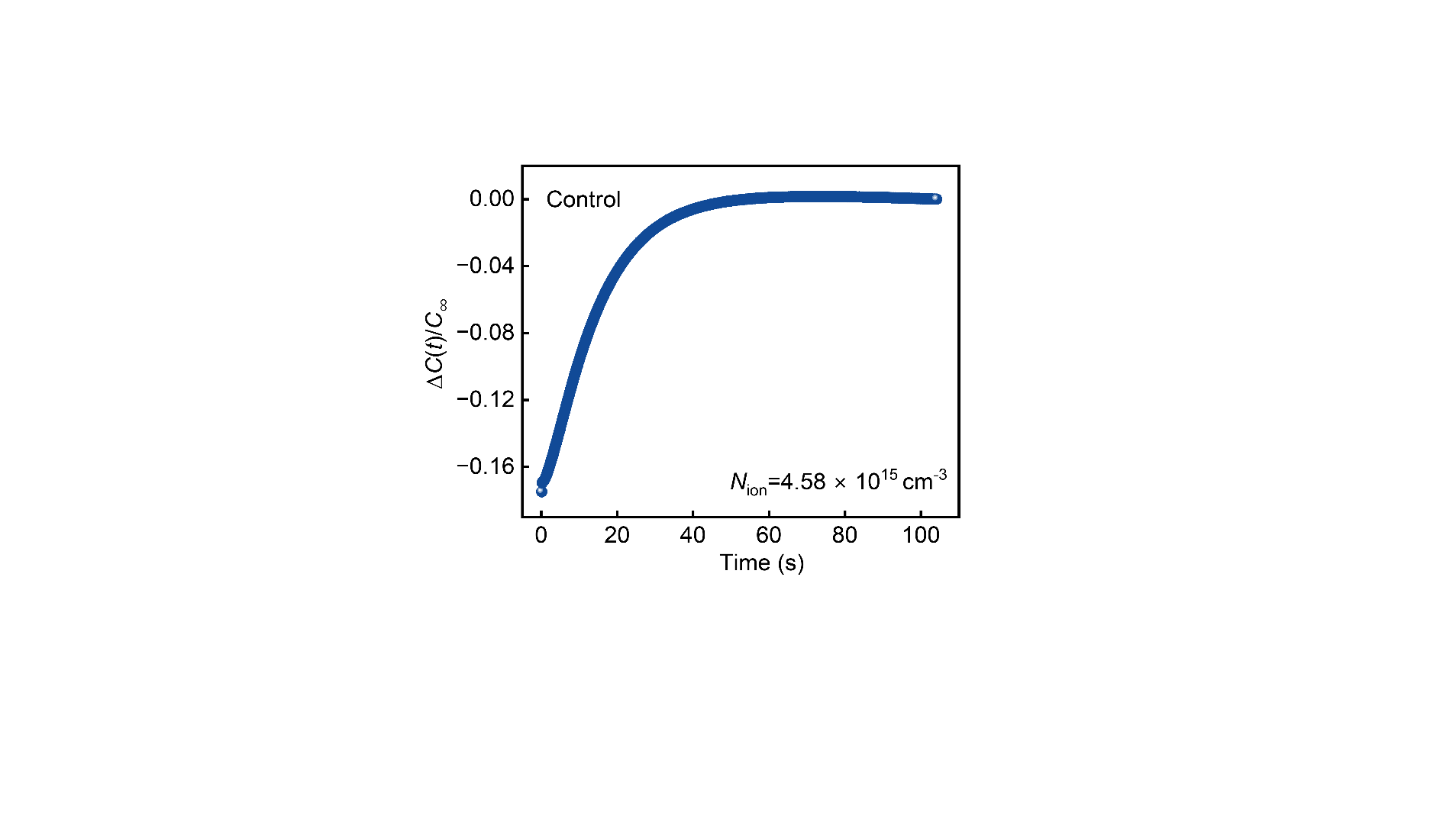


**Figure S16.** Derived density of mobile ions from TID results.


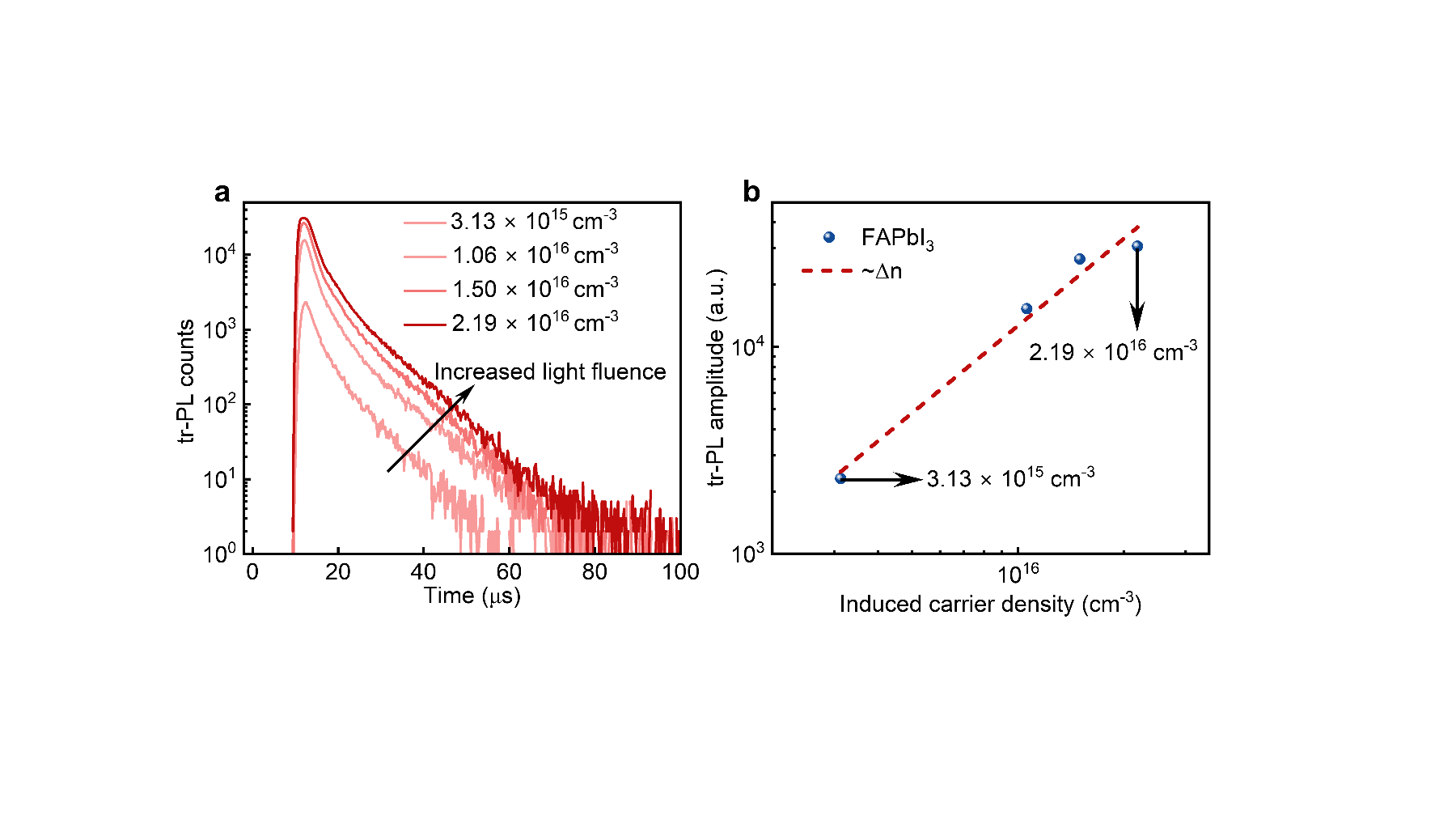


**Figure S17.** a, The results of the tr-PL vs. fluence measurement. b, The dependence of the initial tr-PL amplitude on the induced carrier density.


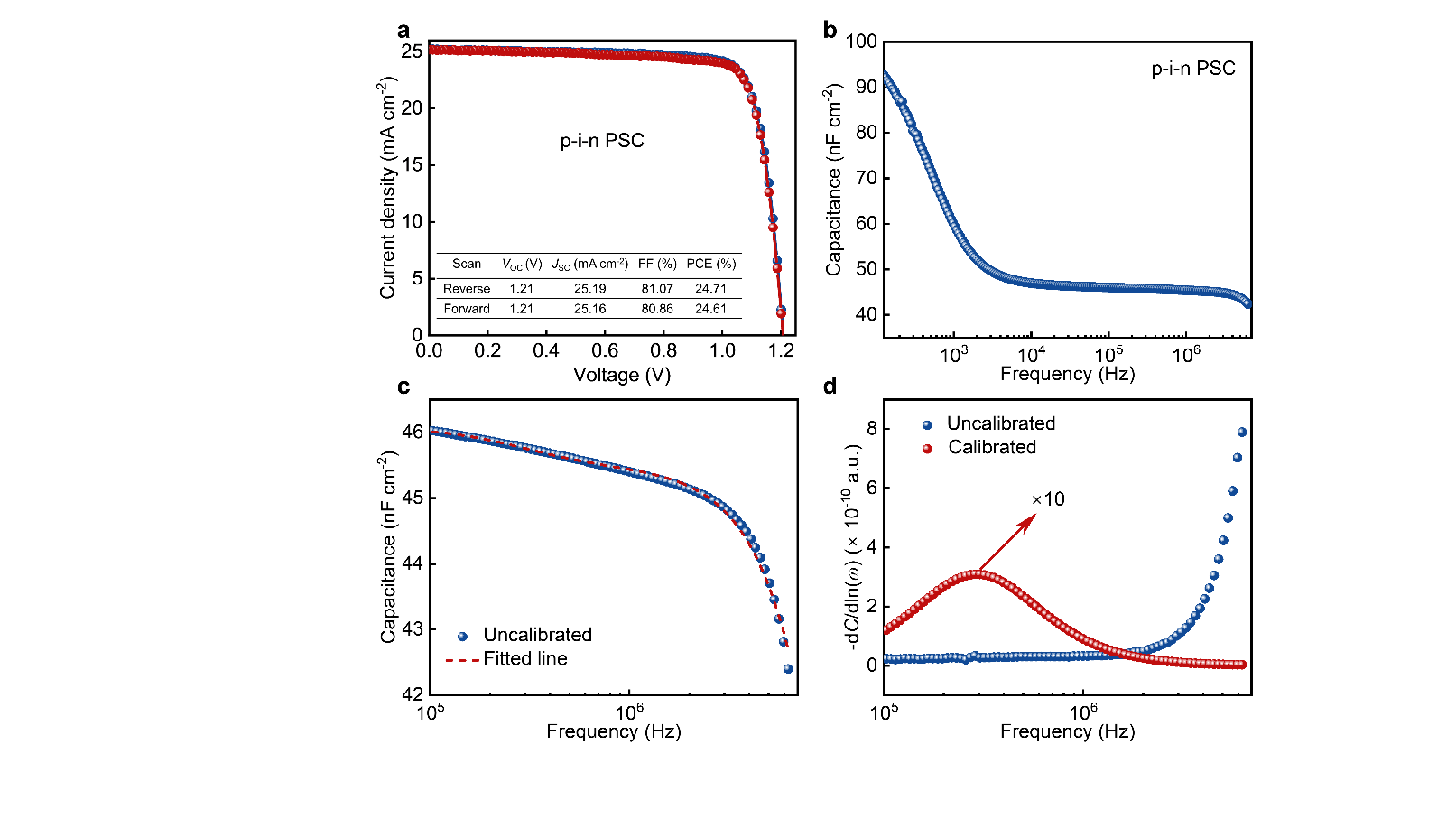


**Figure S18.** a, *J* - *V* curves of the fabricated p-i-n perovskite solar cells. b, *C* – *ω* plot of the fabricated device. c, Numerical fitting of the *C* – *ω* data at the 10^5^ - 10^6^ Hz frequency domain. d, Calibrated and uncalibrated d*C*/dln*ω* – *ω* plot of the fabricated device.


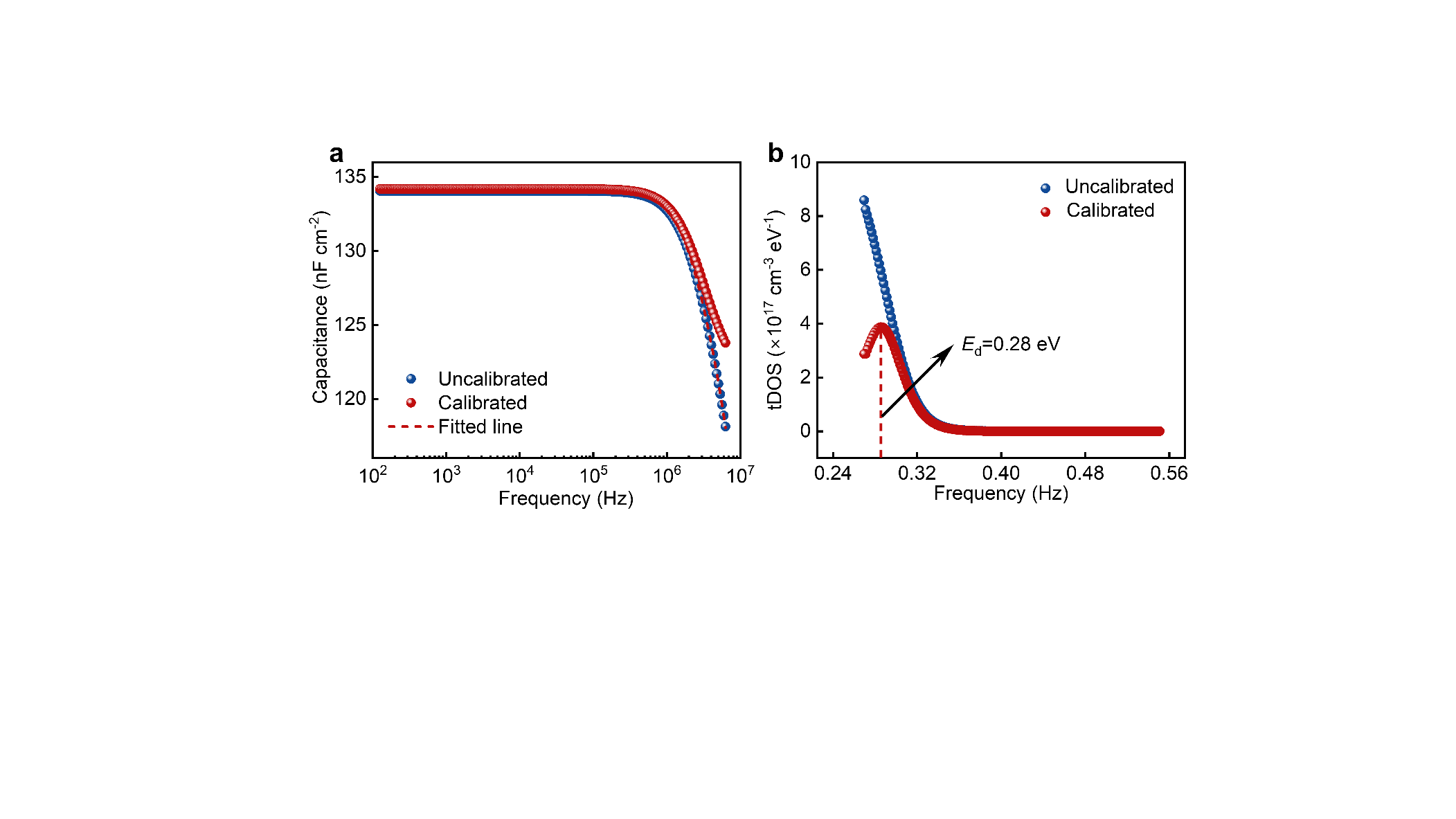


**Figure S19.** a, Calibrated and uncalibrated simulated *C* - *ω* plot. b, Calibrated and uncalibrated simulated tDOS results.

**Note S4 EBIC tests**

In an EBIC experiment, electron beam generated carriers are separated by the built-in potential in the test device which generates a current (EBIC) and can be measured externally by an ammeter^14,15^. The cross-section EBIC sample was prepared by mechanically cracking the PSCs which is a common adopted method for EBIC sample preparation^14,15^. Note that here an accelerating voltage of 5 kV is used to avoid electron beam damage on perovskite films as far as possible. The inclined EBIC curve from the SnO_2_/Perovskite interface to Spiro/Perovskite interface indicates that the fabricated perovskite film is not an intrinsic film. Otherwise, the extracted EBIC will be a flat line across the perovskite films^15-17^. EBIC is proportional to the intensity of the electric field in the space charge region. The highest EBIC close to the SnO_2_/Perovskite interface means that the intensity of the electric field is strongest at the SnO_2_/Perovskite interface, which verifies that the SnO_2_/Perovskite p-n junction is mainly responsible for the charge collection in the fabricated perovskite solar cells^15,18^. Therefore, there is a well-defined depletion region in the fabricated perovskite solar cells, which is formed due to the work function difference between perovskites and SnO_2_. Since SnO_2_ is a well-known n-type material, this result confirms that the fabricated perovskite film is of p-type conductivity.


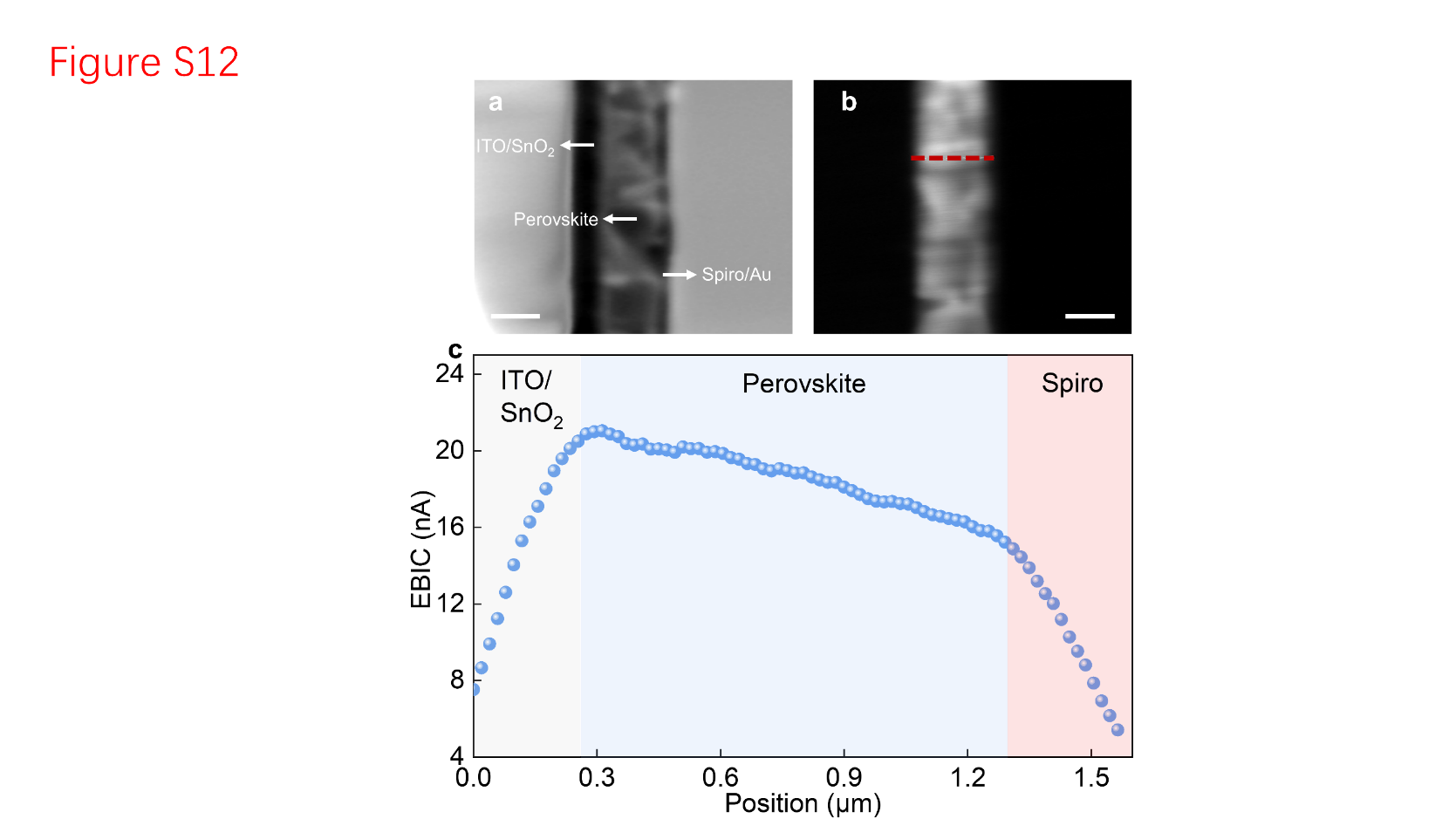


**Figure S20.** (a) A cross-sectional scanning electron microscope image of a PSC with Spiro. The scale bar is 1 μm. (b) The corresponding EBIC mapping image for a PSC with Spiro, where the greyscale stands for the value of EBIC. The scale bar is 1 μm. (c) Extracted EBIC line scan from the EBIC mapping result (b) at the red dash line.

**
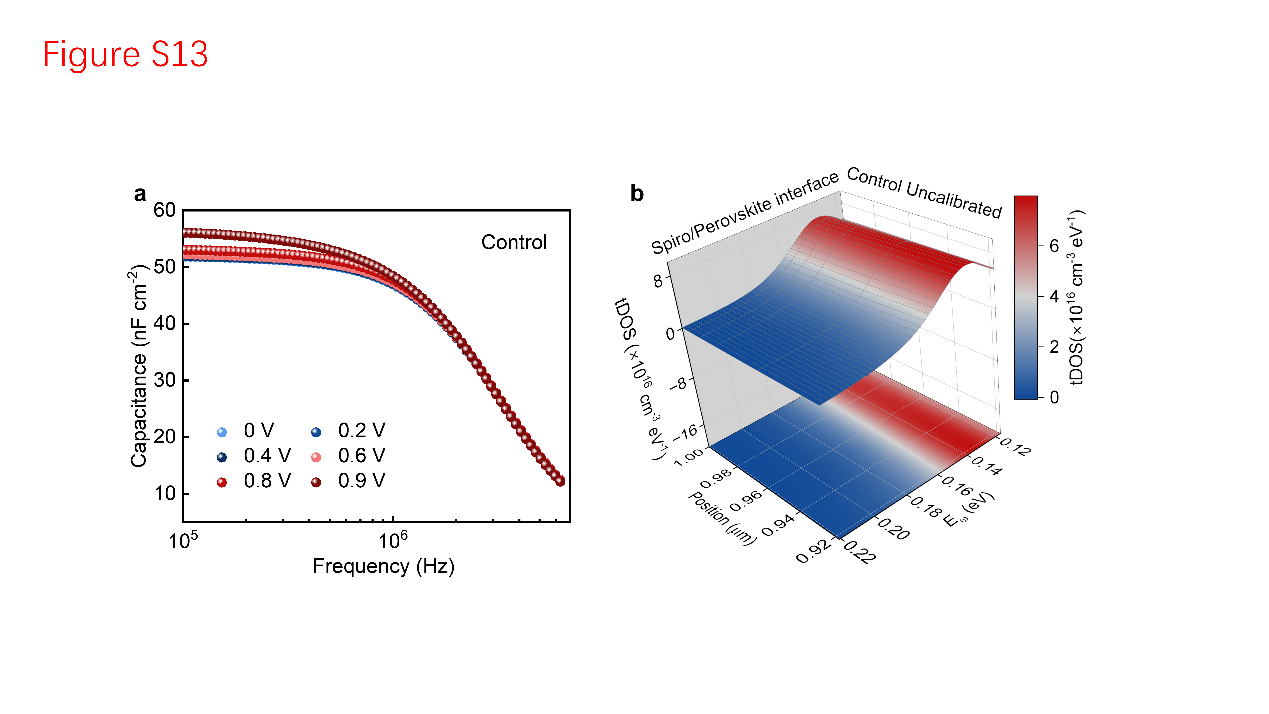
**

**Figure S21.** (a) Raw capacitance data for a PSC without interface passivation. (b) Uncalibrated tDOS of trap band I at different positions in a PSC without interface passivation.


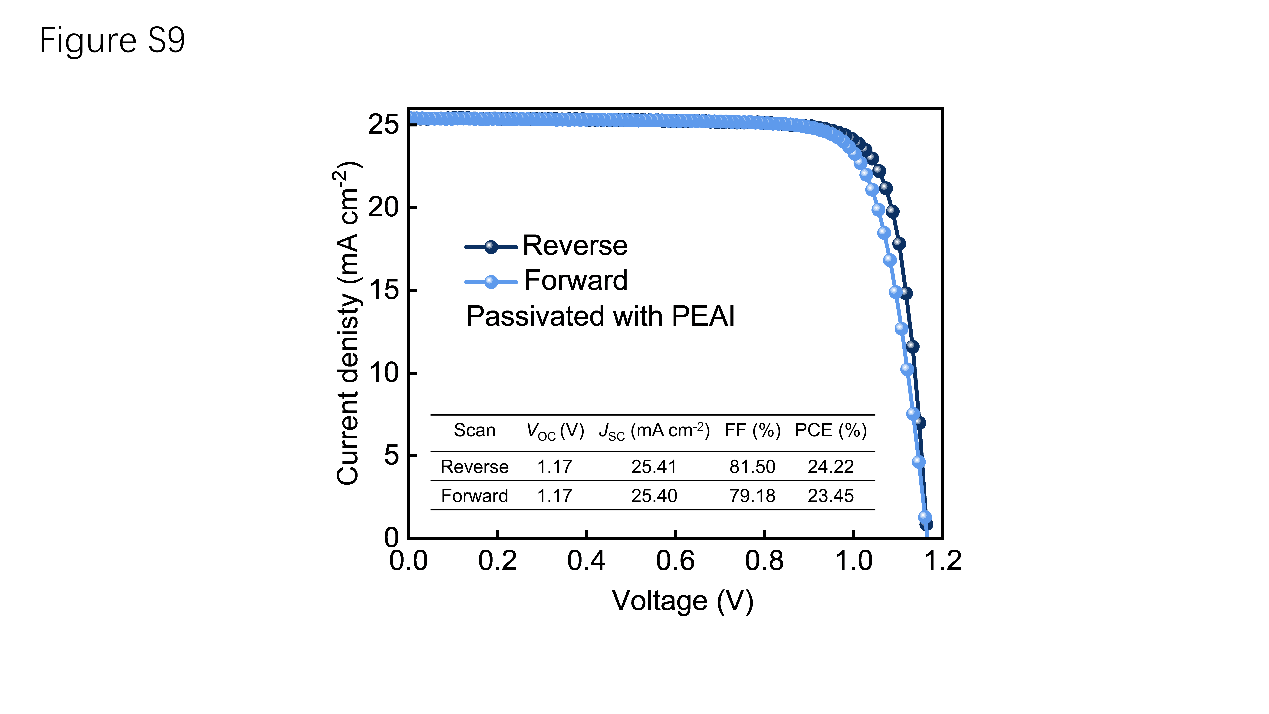


**Figure S22.** *J-V* curves for a representative PSC passivated by PEAI.

**
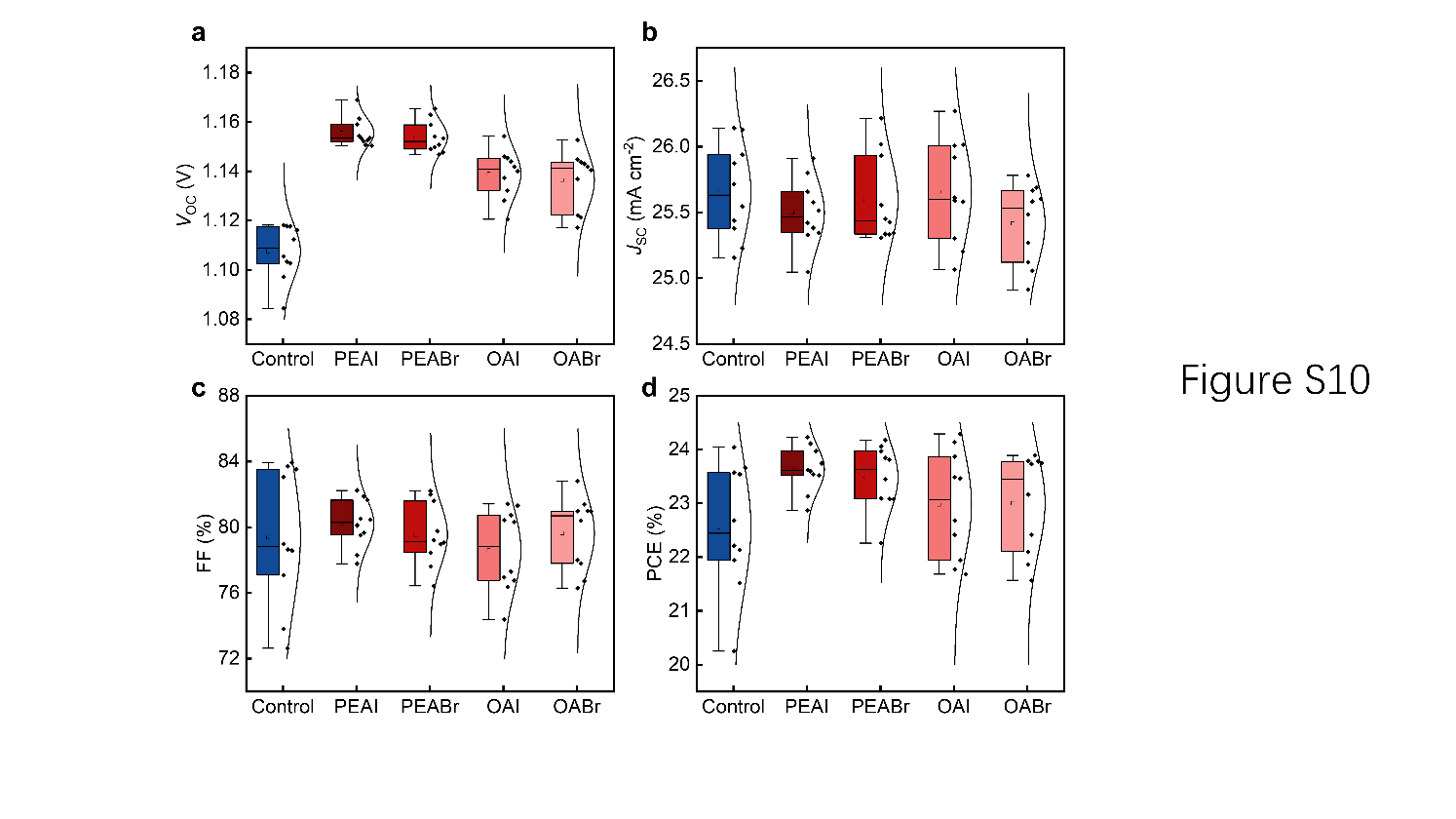
**

**Figure S23.** Parameters of the performance of the fabricated PSCs at reverse scan mode without and with interface passivation by different passivation agencies: (a) *V*_OC_; (b) *J*_SC_; (c) FF; (d) PCE.

**
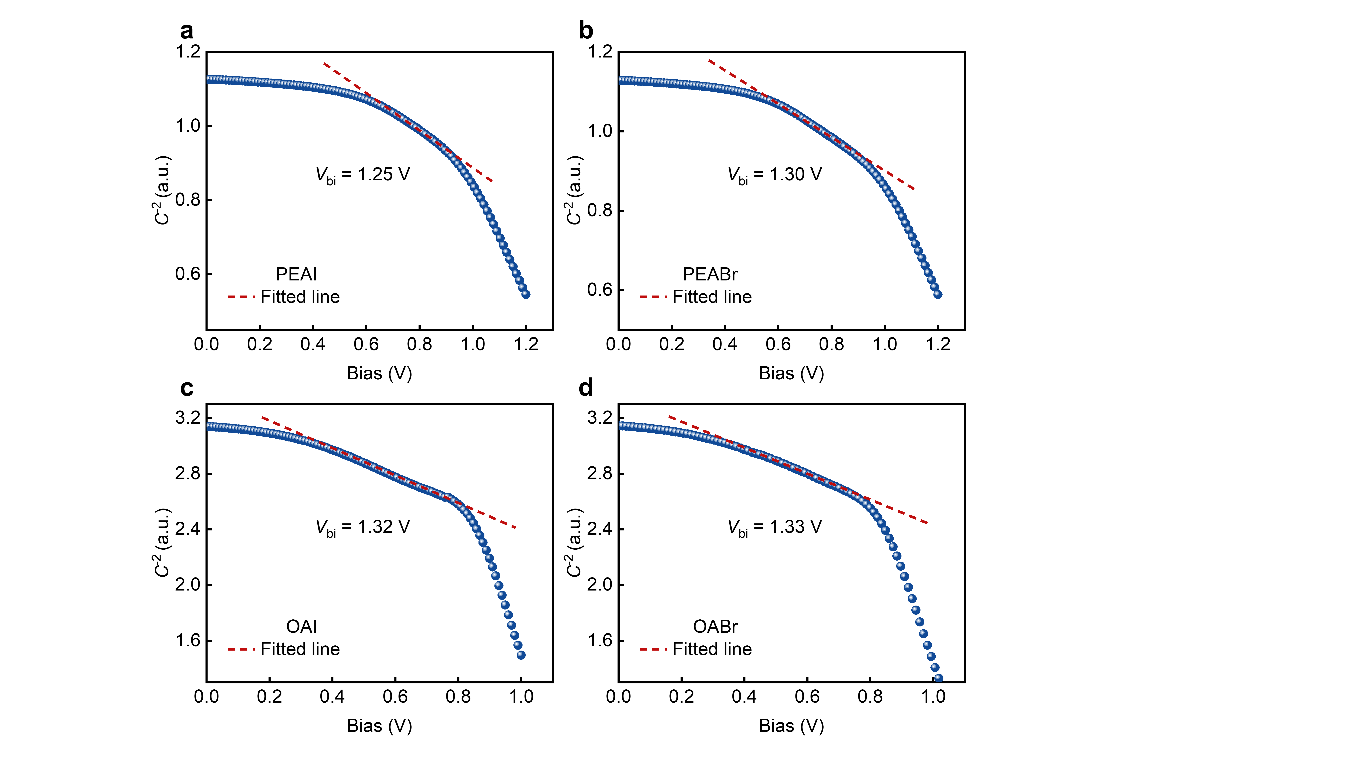
**

**Figure S24.** *C* - *V* curves for PSCs with interface passivation by different passivation agencies: (a) PEAI; (b) PEABr; (c) OAI; (d) OABr.


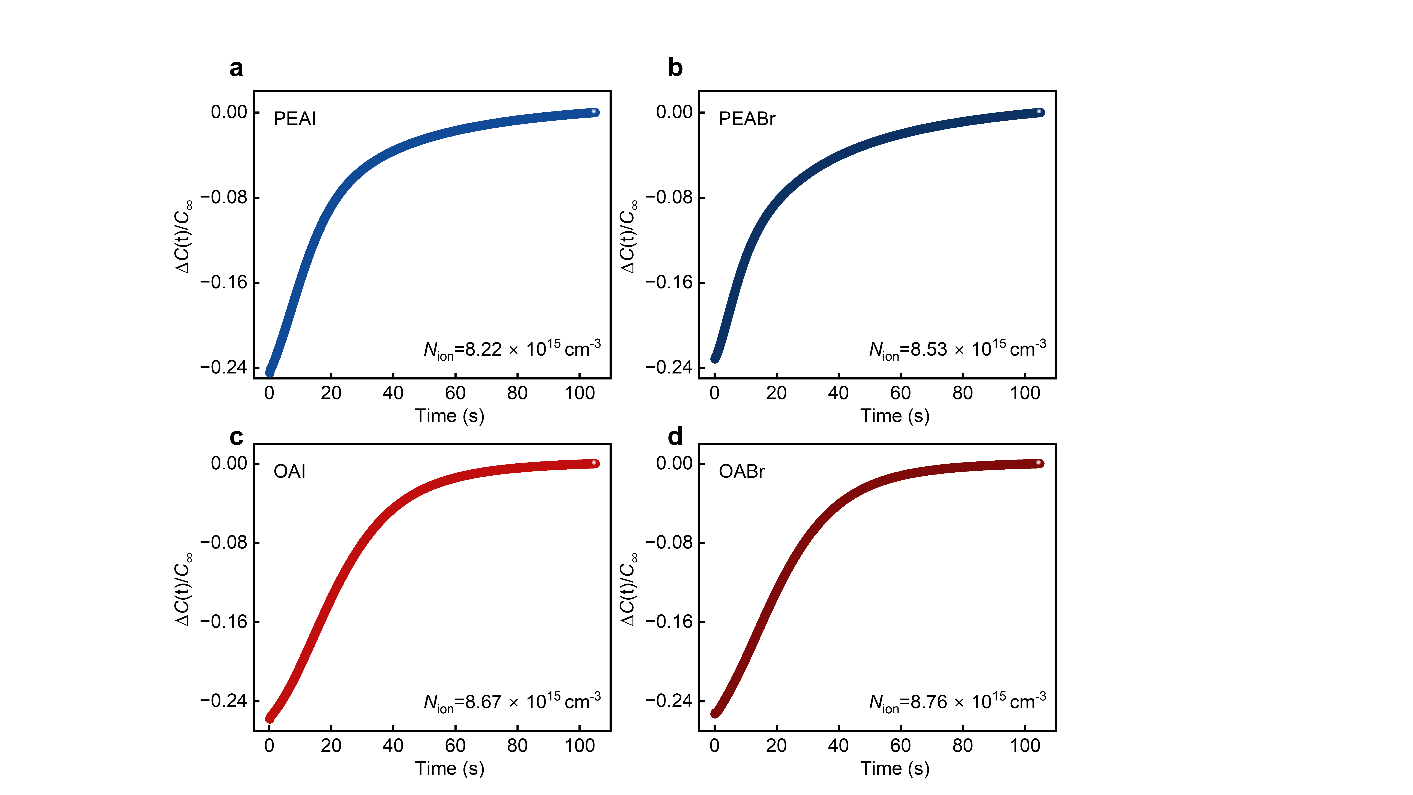


**Figure S25.** Derived density of mobile ions from TID results for PSCs with interface passivation by different passivation agencies: (a) PEAI; (b) PEABr; (c) OAI; (d) OABr.

**Table S5.** Extracted parameters for the calculation of *V*_bi_ of PSCs with interface passivation by different passivation agencies.

| Passivation agencies | *N* (cm^-3^) | *N*_ion_ (cm^-3^) | *N*_app_ (cm^-3^) | *V*_bi_ (V) |
| --- | --- | --- | --- | --- |
| control | 1.31 × 10^16^ | 4.58 × 10^15^ | 8.52 × 10^15^ | 1.30 |
| PEAI | 1.61 × 10^16^ | 7.88 × 10^15^ | 8.22 × 10^15^ | 1.25 |
| PEABr | 1.59 × 10^16^ | 7.37 × 10^15^ | 8.53 × 10^15^ | 1.30 |
| OAI | 1.79 × 10^16^ | 9.23 × 10^15^ | 8.67 × 10^15^ | 1.32 |
| OABr | 1.77 × 10^16^ | 8.94 × 10^15^ | 8.76 × 10^15^ | 1.33 |


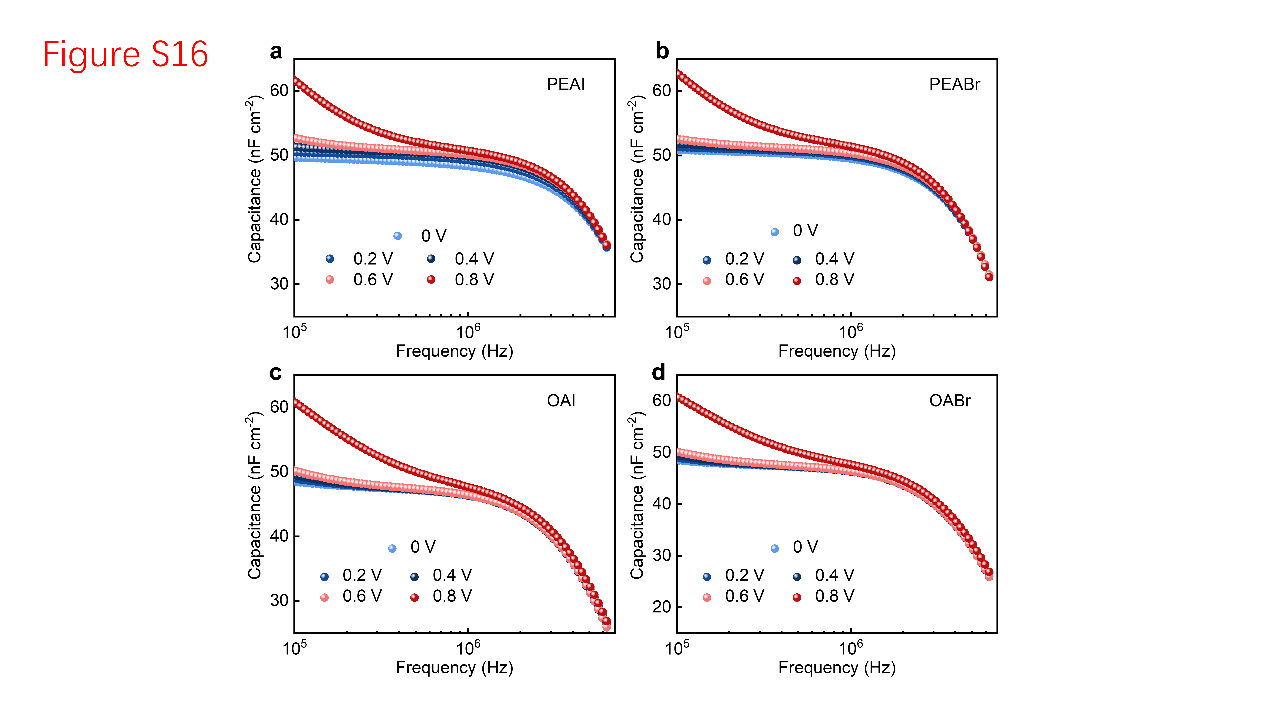


**Figure S26.** Raw capacitance data for PSCs with interface passivation by different passivation agencies: (a) PEAI; (b) PEABr; (c) OAI; (d) OABr.

**
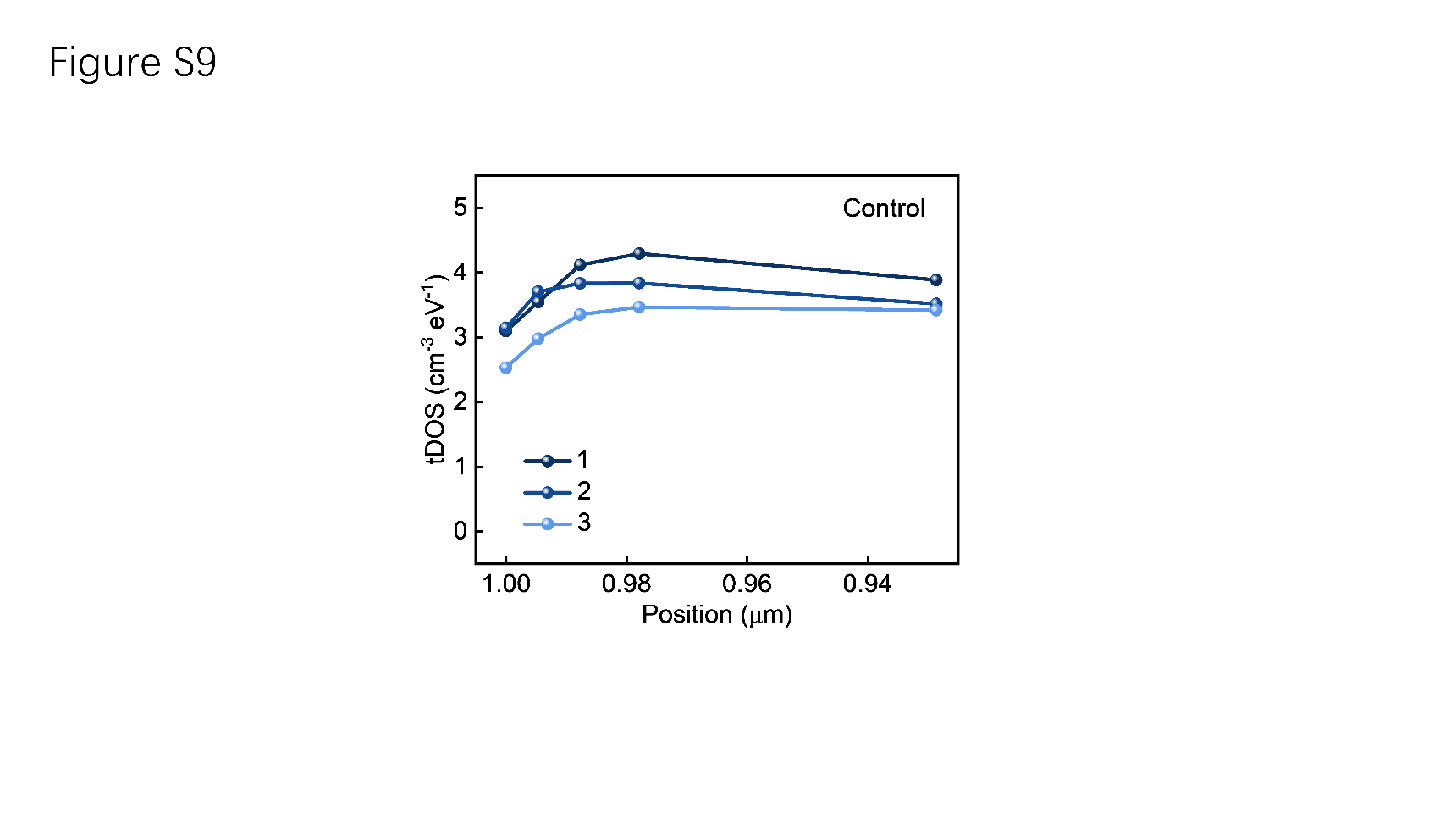
**

**Figure S27.** Calibrated maximum tDOS of trap band I at different positions in three individual PSCs without interface passivation.

**
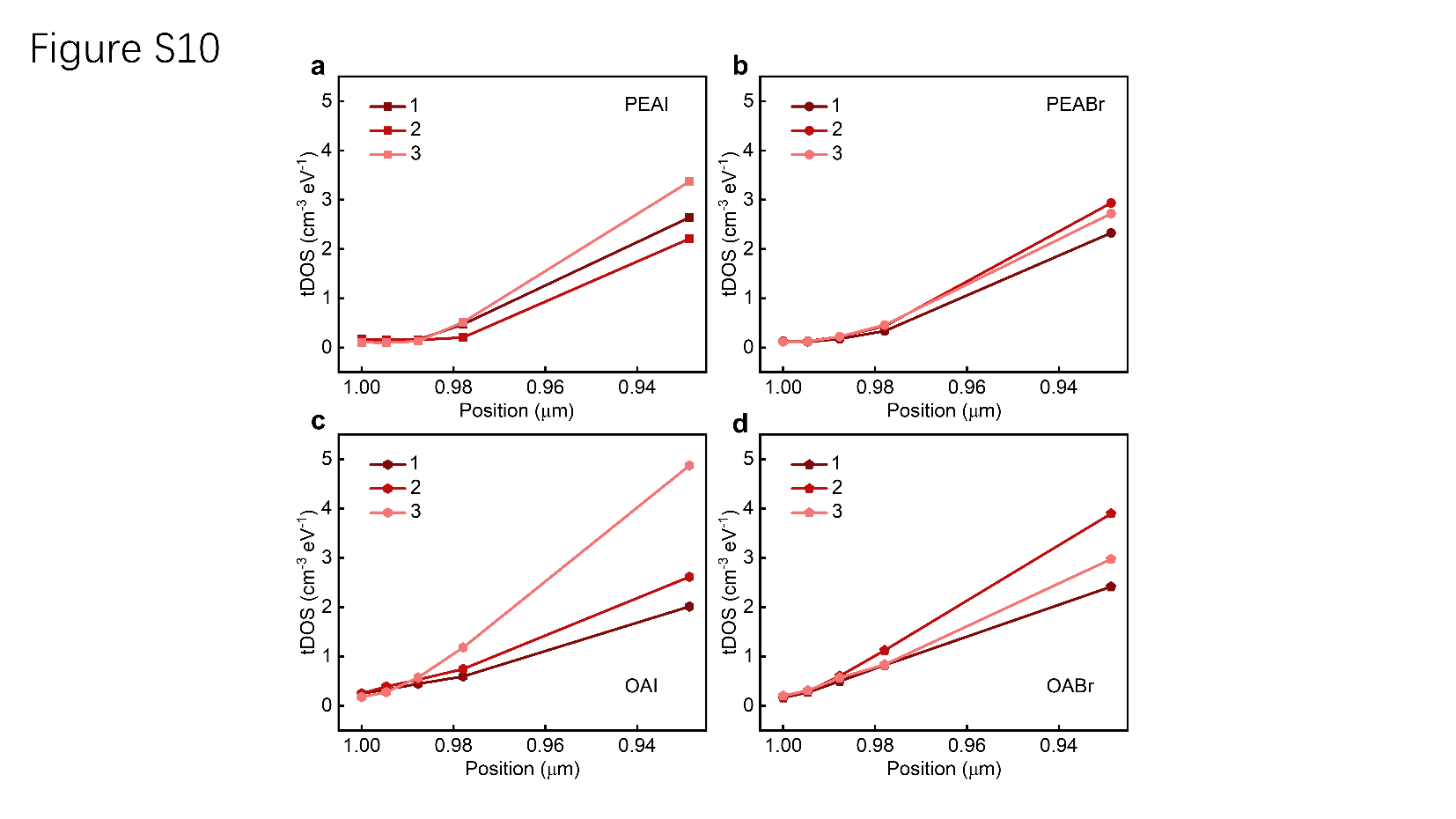
**

**Figure S28.** Calibrated maximum tDOS of trap band I at different positions in three individual PSCs with interface passivation by different passivation agencies: (a) PEAI; (b) PEABr; (c) OAI; (d) OABr.

**References**

1. M. Burgelman, P. Nollet, S. Degrave, *Thin Solid Films* **2000**, 361, 527.

2. M. Fischer, K. Tvingstedt, A. Baumann, V. Dyakonov, *ACS Appl. Energy Mater.* **2018**, 10, 5129.

3. J. Diekmann, F. Peña-Camargo, N. Tokmoldin, J. Thiesbrummel, J. Warby, E. Gutierrez-Partida, S. Shah, D. Neher, M. Stolterfoht, *J. Phys. Chem. Lett.* **2023**, 18, 4200.

4. F. Azri, A. Meftah, N. Sengouga, A. Meftah, *Sol. Energy* **2019**, 181, 372.

5. Y. Raoui, H. Ez-Zahraouy, N. Tahiri, O. E. Bounagui, S. Ahmad, S. Kazim, *Sol. Energy* **2019**, 193, 948.

6. M. H. Futscher, M. K. Gangishetty, D. N. Congreve, B. Ehrler, *J. Chem. Phys.* **2020**, 152, 044202.

7. H. Duan, H. Zhou, Q. Chen, P. Sun, S. Luo, T. Song, B. Boba, Y. Yang, *Phys. Chem. Chem. Phys.* **2015**, 17, 112.

8. S. H. Cho, J. Byeon, K. Jeong, J. Hwang, H. Lee, J. Jang, J. Lee, T. Kim, K. Kim, M. Choi, Y. S. Lee, *Adv. Energy Mater.* **2021**, 11, 2100555.

9. O. Almora, M. García-Batlle, G. Garcia-Belmonte, *J. Phys. Chem. Lett.* **2019**, 10, 3661.

10. S. Ravishankar, Z. Liu, U. Rau, T. Kirchartz, *PRX Energy* **2022**, 1, 013003.

11. M. H. Futscher, J. M. Lee, L. McGovern, L. A. Muscarella, T. Wang, M. I. Haider, A. Fakharuddin, L. Schmidt-Mende, B. Ehrler, *Mater. Horiz.* **2019**, 6, 1497-1503.

12. S. Reichert, Q. An, Y. Woo, A. Walsh, Y. Vaynzof, C. Deibel, *Nat. Commun*. **2020**, 11, 6098.

13. F. Peña-Camargo, J. Thiesbrummel, H. Hempel, A. Musiienko, V. M. Le Corre, J. Diekmann, J. Warby, T. Unold, F. Lang, D. Neher, M. Stolterfoht, *Appl. Phys. Rev.* **2022**, 9, 021409.

14. E. Edri, S. Kirmayer, A. Henning, S. Mukhopadhyay, K. Gartsman, Y. Rosenwaks, G. Hodes, D. Cahen, *Nano. Lett.* **2014**, 14, 1000.

15. E Edri, S. Kirmayer, S. Mukhopadhyay, K. Gartsman, G. Hodes, D. Cahen, *Nat. Commun*. **2014**, 5, 3461.

16. M. Nichterwitz, T. Unold, *J. Appl. Phys.* **2013**, 114, 134504.

17. D. Abou-Ras, T. Kirchartz, *ACS Appl. Energy Mater.* **2019**, 9, 6127.

18. J. Byeon, J. Kim, J. Kim, G. Lee, K. Bang, N. Ahn, M. Choi, *ACS Energy Lett.* **2020**, 8, 2580.
